# Genotype-Environment Interaction Analysis of NQO1, CYP2E1, and NAT2 Polymorphisms and the Risk of Childhood Acute Lymphoblastic Leukemia: A Report From the Mexican Interinstitutional Group for the Identification of the Causes of Childhood Leukemia

**DOI:** 10.3389/fonc.2020.571869

**Published:** 2020-09-21

**Authors:** Aurora Medina-Sanson, Juan Carlos Núñez-Enríquez, Eduardo Hurtado-Cordova, María Luisa Pérez-Saldivar, Anayeli Martínez-García, Elva Jiménez-Hernández, Juan Carlos Fernández-López, Jorge Alfonso Martín-Trejo, Héctor Pérez-Lorenzana, Janet Flores-Lujano, Raquel Amador-Sánchez, Felix Gustavo Mora-Ríos, José Gabriel Peñaloza-González, David Aldebarán Duarte-Rodríguez, José Refugio Torres-Nava, Juan Eduardo Flores-Bautista, Rosa Martha Espinosa-Elizondo, Pedro Francisco Román-Zepeda, Luz Victoria Flores-Villegas, Juana Esther González-Ulivarri, Sofía Irene Martínez-Silva, Gilberto Espinoza-Anrubio, Carolina Almeida-Hernández, Rosario Ramírez-Colorado, Luis Hernández-Mora, Luis Ramiro García-López, Gabriela Adriana Cruz-Ojeda, Arturo Emilio Godoy-Esquivel, Iris Contreras-Hernández, Abraham Medina-Hernández, María Guadalupe López-Caballero, Norma Angélica Hernández-Pineda, Jorge Granados-Kraulles, María Adriana Rodríguez-Vázquez, Delfino Torres-Valle, Carlos Cortés-Reyes, Francisco Medrano-López, Jessica Arleet Pérez-Gómez, Annel Martínez-Ríos, Antonio Aguilar-De los Santos, Berenice Serafin-Díaz, Vilma Carolina Bekker-Méndez, Minerva Mata-Rocha, Blanca Angélica Morales-Castillo, Omar Alejandro Sepúlveda-Robles, Julián Ramírez-Bello, Haydeé Rosas-Vargas, Alfredo Hidalgo-Miranda, Juan Manuel Mejía-Aranguré, Silvia Jiménez-Morales

**Affiliations:** ^1^Departamento de Hemato-Oncología, Hospital Infantil de México Federico Gómez, Secretaría de Salud, Mexico City, Mexico; ^2^Programa de Maestría y Doctorado en Ciencias Médicas de la Facultad de Medicina, Universidad Nacional Autónoma de México (UNAM)Mexico City, Mexico; ^3^Unidad de Investigación Médica en Epidemiología Clínica, Unidad Médica de Alta Especialidad Hospital de Pediatría “Dr. Silvestre Frenk Freund”, Centro Médico Nacional Siglo XXI, Instituto Mexicano del Seguro Social, Mexico City, Mexico; ^4^Laboratorio de Genómica del Cáncer, Instituto Nacional de Medicina Genómica (INMEGEN), Mexico City, Mexico; ^5^Universidad Xochicalco, Campos Tijuana, Tijuana, Mexico; ^6^Facultad de Estudios Superiores Zaragoza, Universidad Nacional Autónoma de México, Mexico City, Mexico; ^7^Servicio de Hematología Pediátrica, Centro Médico Nacional “La Raza”, Hospital General “Gaudencio González Garza”, Instituto Mexicano del Seguro Social (IMSS), Mexico City, Mexico; ^8^Genómica Computacional, Instituto Nacional de Medicina Genómica (INMEGEN), Mexico City, Mexico; ^9^Servicio de Hematología Pediátrica, Centro Médico Nacional “Siglo XXI”, UMAE Hospital de Pediatría “Dr. Silvestre Frenk Freund”, Instituto Mexicano del Seguro Social (IMSS), Mexico City, Mexico; ^10^Servicio de Cirugía Pediátrica, Hospital General “Gaudencio González Garza”, Centro Médico Nacional Siglo XXI (CMN) “La Raza”, Instituto Mexicano del Seguro Social (IMSS), Mexico City, Mexico; ^11^Servicio de Hematología Pediátrica, Hospital General Regional “Carlos McGregor Sánchez Navarro”, Instituto Mexicano del Seguro Social (IMSS), Mexico City, Mexico; ^12^Cirugía Pediátrica del Hospital Regional “General Ignacio Zaragoza”, Instituto de Seguridad y Servicios Sociales de los Trabajadores del Estado (ISSSTE), Mexico City, Mexico; ^13^Servicio de Onco-Pediatria, Hospital Juárez de México, Secretaría de Salud (SS), Mexico City, Mexico; ^14^Servicio de Oncología, Hospital Pediátrico de Moctezuma, Secretaría de Salud de la Ciudad de México (SSCDMX), Mexico City, Mexico; ^15^Servicio de Pediatría, Hospital General de Tláhuac, Secretaría de Salud (SS), Mexico City, Mexico; ^16^Servicio de Hematología Pediátrica, Hospital General de México, Secretaría de Salud (SS), Mexico City, Mexico; ^17^Coordinación Clínica y Servicio de Cirugía pediátrica, Hospital General Regional (HGR) No. 1 “Dr. Carlos Mac Gregor Sánchez Navarro”, Instituto Mexicano del Seguro Social (IMSS), Mexico City, Mexico; ^18^Servicio de Hematología Pediátrica, Centro Médico Nacional “20 de Noviembre”, Instituto de Seguridad y Servicios Sociales de los Trabajadores del Estado (ISSSTE), Mexico City, Mexico; ^19^Jefatura de Enseñanza, Hospital Pediátrico de Iztacalco, Secretaría de Salud de la Ciudad de México (SSCDMX), Mexico City, Mexico; ^20^Jefatura de Enseñanza, Hospital Pediátrico de Iztapalapa, Secretaría de Salud de la Ciudad de México (SSCDMX), Mexico City, Mexico; ^21^Servicio de Pediatría, Hospital General Zona (HGZ) No. 8 “Dr. Gilberto Flores Izquierdo”, Instituto Mexicano del Seguro Social (IMSS), Mexico City, Mexico; ^22^Jefatura de Enseñanza, Hospital General de Ecatepec “Las Américas”, Instituto de Salud del Estado de México (ISEM), Mexico City, Mexico; ^23^Jefatura de Enseñanza, Hospital Pediátrico La Villa, Secretaría de Salud de la Ciudad de México (SSCDMX), Mexico City, Mexico; ^24^Jefatura de Enseñanza, Hospital Pediátrico San Juan de Aragón, Secretaría de Salud (SS), Mexico City, Mexico; ^25^Servicio de Pediatría, Hospital Pediátrico de Tacubaya, Secretaría de Salud de la Ciudad de México (SSCDMX), Mexico City, Mexico; ^26^Coordinación Clínica de Educación e Investigación en Salud, Hospital General de Zona (HGZ) No. 47, IMSS, Mexico City, Mexico; ^27^Servicio de Cirugía Pediátrica, Hospital Pediátrico de Moctezuma, Secretaría de Salud de la Ciudad de México (SSCDMX), Mexico City, Mexico; ^28^Coordinación de Investigación en Salud, Instituto Mexicano del Seguro Social (IMSS), Mexico City, Mexico; ^29^Pediatría, Hospital Materno-Pediátrico de Xochimilco, Secretaría de Salud de la Ciudad de México (SSCDMX), Mexico City, Mexico; ^30^Jefatura de Enseñanza, Hospital Pediátrico de Coyoacán, Secretaría de Salud de la Ciudad de México (SSCDMX), Mexico City, Mexico; ^31^Coordinación Clínica y Pediatría del Hospital General de Zona 76, Instituto Mexicano del Seguro Social (IMSS), Mexico City, Mexico; ^32^Coordinación Clínica y Pediatría del Hospital General de Zona 68, Instituto Mexicano del Seguro Social (IMSS), Mexico City, Mexico; ^33^Coordinación Clínica y Pediatría del Hospital General de Zona 71, Instituto Mexicano del Seguro Social (IMSS), Mexico City, Mexico; ^34^Pediatría, Hospital General Dr. Darío Fernández Fierro, Instituto de Seguridad y Servicios Sociales de los Trabajadores del Estado (ISSSTE), Mexico City, Mexico; ^35^Coordinación Clínica y Servicio de Pediatría, Hospital General Regional (HGR) No. 72 “Dr. Vicente Santos Guajardo”, Instituto Mexicano del Seguro Social (IMSS), Mexico City, Mexico; ^36^Cirugía Pediátrica del Hospital Regional “General Ignacio Zaragoza”, Instituto de Seguridad y Servicios Sociales de los Trabajadores del Estado (ISSSTE), Mexico City, Mexico; ^37^Coordinación Clínica y Pediatría del Hospital General de Zona 98, Instituto Mexicano del Seguro Social (IMSS), Mexico City, Mexico; ^38^Coordinación Clínica y Pediatría del Hospital General de Zona 57, Instituto Mexicano del Seguro Social (IMSS), Mexico City, Mexico; ^39^Hospital de Infectología “Dr. Daniel Méndez Hernández”, “La Raza”, Instituto Mexicano del Seguro Social (IMSS), Unidad de Investigación Médica en Inmunología e Infectología, Mexico City, Mexico; ^40^Unidad de Investigación Médica en Genética Humana, UMAE Hospital de Pediatría “Dr. Silvestre Frenk Freund”, Centro Médico Nacional “Siglo XXI”, Instituto Mexicano del Seguro Social (IMSS), Mexico City, Mexico; ^41^Unidad de Investigación, Hospital Juárez de México, México City, Mexico

**Keywords:** acute lymphoblastic leukemia, ancestry informative markers, NAT2 polymorphisms, association study, Mexican mestizos

## Abstract

**Background:** Acute lymphoblastic leukemia (ALL) is the main type of cancer in children. In Mexico and other Hispanic populations, the incidence of this neoplasm is one of the highest reported worldwide. Functional polymorphisms of various enzymes involved in the metabolism of xenobiotics have been associated with an increased risk of developing ALL, and the risk is different by ethnicity. The aims of the present study were to identify whether *NQO1, CYP2E1*, and *NAT2* polymorphisms or some genotype-environmental interactions were associated with ALL risk in Mexican children.

**Methods:** We conducted a case-control study including 478 pediatric patients diagnosed with ALL and 284 controls (children without leukemia). Ancestry composition of a subset of cases and controls was assessed using 32 ancestry informative markers. Genetic-environmental interactions for the exposure to hydrocarbons were assessed by logistic regression analysis.

**Results:** The polymorphisms rs1801280 (OR 1.54, 95% CI 1.21–1.93), rs1799929 (OR 1.96, 95% CI 1.55–2.49), and rs1208 (OR 1.44, 95% CI 1.14–1.81) were found to increase the risk of ALL; being the risks higher under a recessive model (OR 2.20, 95% CI 1.30–1.71, OR 3.87, 95% CI 2.20–6.80, and OR 2.26, 95% CI 1.32–3.87, respectively). Gene-environment interaction analysis showed that *NAT2* rs1799929 TT genotype confers high risk to ALL under exposure to fertilizers, insecticides, hydrocarbon derivatives, and parental tobacco smoking. No associations among *NQO1, CYP2E1*, and ALL were observed.

**Conclusion:** Our study provides evidence for the association between *NAT2* polymorphisms/gene-environment interactions, and the risk of childhood ALL in Mexican children.

## Introduction

Acute lymphoblastic leukemia (ALL) represents the most frequent type of cancer in pediatric population worldwide. Additionally, Mexico and other Hispanic populations have one of the highest incidence and mortality rates for this cancer around the globe (1, 8). The etiology is unclear; however, it has been recognized that the interaction between genetic and environmental factors may play a role in the development of the disease. It has been suggested that enzymes involved in the metabolism of carcinogenic agents are associated with ALL susceptibility; as well, it could explain the differences in survival rates reported across populations (9–11).

Hydrocarbons, parental smoking, alcohol drinking, pesticides, and air pollution have been reported as the main type of xenobiotics related with an increased risk of developing ALL in children (12–16). Afterwards, these compounds enter into the body, they undergo biotransformation and elimination processes by xenobiotic metabolizing enzymes (2, 17, 18). Phase I enzymes are responsible for catalyzing reactions of hydroxylation, reduction and oxidation of xenobiotics, ultimately, transforming these into more toxic compounds. Phase II enzymes participate in conjugation processes, such as glucuronidation, acetylation, and methylation, converting the metabolites into non-reactive and water-soluble products which could be more easily eliminated from the body (18).

Cytochrome P450 Family 2 Subfamily E Member 1 (CYP2E1) are phase I enzymes, whereas, NAD(P)H quinone oxidoreductase 1 (NQO1) and Arylamine N-acetyltransferase 2 (NAT2) are phase II enzymes, which are commonly implicated in xenobiotics metabolism (2). It is well-recognized that genetic polymorphisms could influence the activity of these enzymes by affecting at a transcription level (CYP2E1), enzymes activity (NQO1) or at a protein level (NAT2) (2, 19). In addition, diverse studies have reported that polymorphisms in genes encoding xenobiotic metabolizing enzymes could increase the risk of developing childhood ALL, early relapse, chemotherapy-related toxicity, treatment resistance and have been associated with lower survival rates in patients with this type of cancer (2, 17). However, the results are inconsistent across populations suggesting the possibility that susceptibility alleles could be associated with ALL in a race-specific manner, as it has been reported for other ALL-predisposing genetic polymorphisms (20, 21).

The aims of the present study were to identify whether *NQO1, CYP2E1*, and *NAT2* polymorphisms, or some genotype-environmental interactions, were associated with ALL risk in Mexican children. As well, to provide a picture of the genetic ancestry and admixture patterns of the patients with ALL from central Mexico.

## Materials and Methods

### Subjects

A case-control study was conducted by the Mexican Interinstitutional Group for the Identification of the Causes of Childhood Leukemia (MIGICCL), from August 1, 2014, to July 31, 2016. Cases and controls were younger than 17 years, residents of the Metropolitan Area of Mexico City.

#### Cases

Cases were recruited from eight public hospitals of Mexico City in which an estimated 69.9% of children with leukemia who reside in Mexico City are attended. Acute lymphoblastic leukemia diagnosis was established according to clinical features, and bone marrow aspirate findings: cell morphology, immunophenotype, and genetics, as defined by the 2008 WHO classification of lymphoid neoplasms Children with Down syndrome were excluded from the analysis. Case registration required that trained personnel were assigned to each participating hospital to identify incident cases of leukemia through reviews of clinical charts. Afterwards, parents were approached and invited to participate.

The participating public hospitals and health institutions were: (1) Hospital de Pediatría, “Dr. Silvestre Frenk Freund” Centro Médico Nacional (CMN) “Siglo XXI”, Instituto Mexicano del Seguro Social (IMSS); (2) Hospital Infantil de México Federico Gómez, Secretaría de Salud (SS); (3) Hospital General “Dr. Gaudencio González Garza”, CMN “La Raza”, IMSS; (4) Hospital General Regional “Dr. Carlos McGregor Sánchez Navarro”, IMSS; (5) Hospital Juárez de México, SS; (6) Hospital Pediátrico de Moctezuma, Secretaria de Salud de la Ciudad de México (SSCDMX); (7) Hospital General de México, SS; and (8) Hospital CMN “20 de Noviembre”, Instituto de Seguridad Social al Servicio de los Trabajadores del Estado (ISSSTE). For this study the sample size was calculated with 250 cases and 250 controls, this being sufficient for obtaining a power of 80% to identify the associations. In parallel to this study the inclusion of cases continued for assessing the prognostic value of the *NQO1, CYP2E1*, and *NAT2* polymorphisms studied here on ALL survival. Those cases were also included in the present work for increasing the precision of the assessed interactions.

#### Controls

The controls were selected from second-level hospitals of the same health institution (IMSS, SS, SSCDMX, and ISSSTE) that referred the children with ALL to the third-level care hospitals. The controls were children without leukemia who were treated at different hospital departments, such as ambulatory surgery, pediatrics, ophthalmology and orthopedic outpatient clinics, and the emergency room. Children with diagnoses of neoplasms, hematological diseases, allergies, infections, and congenital malformations were not selected as controls. The interviewers were provided with a list of characteristics of individual leukemia cases, and charged with identifying one control per case according to sex, age (±18 months), and health institution. When more than one control per case met the matching criteria, the control that was closest in age to the case was selected. If the controls were of the same age, one control was selected randomly (by tossing a coin). Moreover, if no control of the same sex was found after three visits to the same hospital, a control matched by age (±18 months) was selected.

Trained personnel were assigned to each of the second-level hospitals to search for controls who fulfilled the selection criteria. When a control was identified, parents were invited to participate.

The Ethics and National Committee of Scientific Research approved this study with number R-2013-785-062. Additionally, we obtained approval by the Institutional Scientific Review Committees of each health institution to carry out the study.

### Study Overview

The interview was carried out using the same questionnaire for cases and controls. This questionnaire was previously standardized and adapted from the questionnaire module of the National Cancer Institute. General information obtained from the interviews included the child's history, socioeconomic status (SES), demographic characteristics, and the parents information (age at pregnancy, educational level, etc.).

Study variables were child's sex, age, and birthweight (<3,500 and ≥3,500 grams), family history of cancer, maternal years of education, after birth home exposure to fertilizers, insecticides, and other hydrocarbon derivatives (benzene, solvents, glues, petroleum products, etc.), and parental tobacco smoking.

For the exposure of the parents to tobacco smoking, the information was obtained from the in-person interview and it was assessed in the four exposure periods: (1) pre-conceptionally, (2) during pregnancy, (3) during lactation, and (4) the last year before leukemia diagnosis or interview in cases and controls. The number of cigarettes smoked during each exposure period was calculated. Parental smoking was classified into three groups: (1) when both parents were lifelong non-smokers or ex-smokers, who stopped smoking more than 1 year before the birth of the index child, (2) when either parent smoked <5 cigarettes a day, and (3) when at least one parent smoked 6 or more cigarettes daily. Passive smoking in the child was considered when at least one of the parents smoked in the presence of the child for at least three times a week during the year before diagnosis/interview in cases and controls, respectively.

Parental years of education were used as an indicator of SES as it has been previously used by the Childhood Leukemia International Consortium (CLIC) (0–9 years, 9.1–12.9 years [reference category], ≥13 years of education).

### DNA Extraction

Genomic DNA from saliva or peripheral blood was obtained according to the ORAGENE Purification Kit (DNA Genotek Inc., ON, Canada) and the Gentra Kit (Gentra Systems Inc, Minneapolis, MN) manufacturer's instructions, respectively. DNA purity and concentration were determined by sypectrofotometry (Nanodrop-1000).

### Genotyping of NQ01, CYPE2, and NAT2 SNPs

Genotyping of rs1800566 (*NQ01*), rs3813867 (*CYPE2*) and rs1041983 (C282T), rs1801280 (T341C), rs1799929 (C481T), rs1799930 (G590A), rs1208 (A803G), and rs1799931 (G857A) from *NAT2* SNPS was performed using the 5′exonuclease technique and TaqMan MGB chemistry in a QuantStudio 5 system according to the manufacturer's instructions (Thermofisher, Foster City, CA). TaqMan probes used were C___2091255_30 (rs1800566), C___2431875_10 (rs3813867), C___8684085_20 (rs3813867), C___1204093_20 (rs1801280), C___1204092_20 (rs1799929), C___1204091_10 (rs1799930), C____572769_20 (rs1208). Each polymerase chain reaction (PCR) reaction contained 25 ng of genomic DNA, 2.5 ul of TaqMan master mix, 0.0625 ul of 40X assay mix, and ddH_2_O up to a final volume of 5 ul. The PCR protocol included denaturing at 95°C for 10 min, followed by 40 cycles of denaturing at 95°C for 15 s and annealing and extension at 60°C for 1 min. The genotype of each sample was assigned automatically by measuring the allele-specific fluorescence using QuantStudio Desing and Analysis software 5 for allelic discrimination (Applied Biosystems, Foster City, CA). The overall genotype call rate was 99.8%, and a subset of randomized samples were genotyped in duplicate and showed 100% reproducibility.

### Ancestry Informative Markers Selection and Ancestry Composition Analysis

To know the ancestry composition of the ALL pediatric cases, and to account for the effects of population stratification in our case-control association study, we analyzed a subset of ALL cases and controls using a panel of 32 single nucleotide polymorphisms (SNPs) as ancestry informative markers (AIMs). The SNPs chosen for inclusion were based on AIMs sets' that has been validated in a large group of Mexican subjects. This panel included 1 to 2 SNPs in all chromosomes, with the exception of chromosome 18 (22). ALL cases and controls were paired by gender, age, and living area in Mexico-City. To estimate individual and global ancestry we used the STRUCTURE software considering the European and Native-American (NA) populations as the two main contributors to the largest Mexican ethnic group (Mexican-Mestizo) (3). Principal component analysis (PCA) was used to infer population structure within our samples (23).

### Association and Gene-Gene Interaction Analyses

The polymorphisms *NQO1* (rs1800566), *CYP2E1* (rs3813867), and *NAT2* [rs1041983 (C282T), rs1801280 (T341C), rs1799929 (C481T), rs1799930 (G590A), rs1208 (A803G), and rs1799931 (G857A)] were selected based on previous results in leukemia where they were the most consistently associated with leukemia risk (2, 11, 24–26). Association among alleles/genotypes and ALL was tested by comparing cases and controls. There was 99% concordance of randomly repeated samples during the genotyping. Bonferroni correction test was applied to data, then, the p-value for statistical significance varied according to the number of parameters. In addition, all SNPs were evaluated under the co-dominant, dominant, and recessive genetic models using the FINETTI program (http://ihg.gsf.de/cgicbin/hw/hwa1.pl). Linkage disequilibrium (LD) and haplotyping of *NAT2* SNPs were assessed by using the haploview software (27). The Multifactor Dimensionality Reduction (MDR) interaction model was generated by multifactor dimensionality reduction software (V 3.0.2) (28). Results were considered to be significant at *p* ≤ 0.05°.

### NAT2 Acetylator Frequencies Analysis

Patients were phenotyped as rapid acetylators and slow acetylators. Haplotypes containing the wild-type (WT) *NAT2* alleles [rs1041983 (C282T), rs1801280 (T341C), rs1799929 (C481T), rs1799930 (G590A), rs1208 (A803G), and rs1799931], named NAT2^*^4, were classified as a rapid acetylator phenotype. Haplotypes containing more than one mutant allele were identified as slow acetylators and the remainder as an intermediate acetylator phenotype, using the NAT2PRED web-server (http://nat2pred.rit.albany.edu) (29). *NAT2* alleles were identified by using http://nat.mbg.duth.gr/Human%20NAT2%20alleles_2013.htm.

### Evaluation of Interactions Among Polymorphisms and Exposure to Hydrocarbons

To search for gene-environment interactions we used the MDR approach, since this program incorporates information from several *loci* and environmental factors to identify combinations of both factors that are associated with the risk of a disease (30).

### Statistical Analysis

Analyses were performed using SPSS version 21 (IBM Statistical Package for the Social Sciences, Inc., Chicago, IL, USA). Descriptive analyses were conducted. Odds ratio (OR) and 95% confidence intervals (95% CI) were estimated by unconditional logistic regression. The logistic regression model was constructed as follows: (1) the overall interaction in the model was evaluated, (2) it was assessed whether study variables had a global confounding effect, those variables that, in the stratified analysis the ORs prior to (crude OR) and after stratifying (aOR) showed a difference between the two ORs >10%. These variables alongside with the matching variables (child's sex, age, and health institution), were included in the multivariate analyses. As a result, the most parsimonious model included: child's sex, age, paternal education level, maternal education level, maternal age at pregnancy, active smoking of the mother after birth, alcoholism of the mother before pregnancy, family cancer history, health institution, birthweight, maternal exposure to hydrocarbons at home during pregnancy, X-rays exposure during pregnancy, alcoholism of the father before pregnancy, and active smoking of the father after birth. Accordingly, the adjusted ORs (aOR) were calculated.

Hardy-Weinberg Equilibrium (HWE) test was performed using the FINETTI program (http://ihg.gsf.de/cgicbin/hw/hwa1.pl). Alleles and genotypes frequencies were compared among groups by using chi-square and Fisher's exact test, when appropriate, which are implemented in the STATCALC program (Epi Info v.6.02 software, Centers for Disease Control and Prevention, Atlanta, GA). The level of significance was set at 5%. To evaluate ancestry composition of our sample, STRUCTURE software was used assuming two populations (European and NA), and each analysis was performed at least three times using >100,000 replicates and 20,000 burn-in cycles under admixed model. To determine the statistical power of our study, we used Quanto software (http://hydra.usc.edu/gxe) accounting minor allele frequency (MAF) of all SNPs in the control the group; likewise, considering a recessive genetic model, and odd ratio (OR) of 2.0, the prevalence of ALL in Mexican children, and the sample size.

## Results

### Patient Demographic Data

A total of 469 cases were diagnosed with ALL in participant hospitals during the study period. In addition, a total of 285 controls were recruited. Demographic, clinical features and exposure data of the study population are displayed in Supplementary Table 1. The following variables: child's age, parental education level, parental smoking, alcohol drinking by the father, and maternal exposure to hydrocarbons at home showed statistically significant differences between the total of cases with ALL and controls. Cases had a less proportion of active smoking by the mother after birth, alcohol drinking by both parents before pregnancy, and maternal exposure to hydrocarbons at home before pregnancy, but higher proportion of maternal exposure to hydrocarbons at home during pregnancy than controls (Supplementary Table 1). The associations when 279 cases were analyzed and then, when all the available 469 cases were included, were very similar (Supplementary Table 2). It was therefore decided to include the total number of cases in subsequent analyses.

### *NAT2* Polymorphisms Are Associated With Acute Lymphoblastic Leukemia

Except for the rs1041983 (C282T) and rs1799931 (G857A), the remaining SNPs were found in HWE in the control population. The association analysis between individual SNPs and ALL are described in Table 1. We documented statistically significant differences in the distribution of rs1801280 (T341C) (OR 1.54, 95% CI 1.12–1.95), rs1789929 (C481T) (OR 1.95, 95% IC 1.53–2.47), and rs1208 (A803G) (OR 1.47, 95% IC 1.175–1.86) *NAT2* SNPs.

**Table 1:** Genotype and alleles frequency of *NAT2, NQ01*, and *CYP2E1* polymorphisms in children with acute lymphoblastic leukemia and controls.

**Gene**	**SNP**	**Genotypes/Alleles**	**Cases**	**Controls**	**OR (95% CI)**	**aOR (95% CI)**
			**469**	**285**		
			***n* (%)**	***n* (%)**		
NAT2^*^	rs1041983 (C282T)	CC (ref.)	270 (57.7)	145 (51.4)	—	—
		CT	152 (32.5)	102 (36.2)	0.80 (0.58–1.10)	0.73 (0.49–1.09)
		TT	46 (9.8)	35 (12.4)	0.71 (0.43–1.14)	0.56 (0.30–1.07)
		C	692 (73.9)	392 (69.5)	—	—
		T	244 (26.1)	172 (30.5)	0.80 (0.63–1.01)	
	rs1801280 (T341C)	TT (ref.)	220 (47.0)	165 (58.5)	—	—
		TC	182 (38.9)	95 (33.7)	**1.44 (1.04–1.98)**	**1.70 (1.14–2.53)**
		CC	66 (14.1)	22 (7.8)	**2.25 (1.33–3.80)**	**2.03 (1.08–3.82)**
		T	622 (76.5.0)	425 (75.4)	—	—
		C	314 (33.5.0)	139 (24.6)	**1.54 (1.12–1.95)**	
	rs1799929 (C481T)	CC (ref.)	203 (43.4)	167 (59.4)	—	—
		CT	186 (39.7)	96 (34.2)	**1.59 (1.16–2.20)**	**1.93 (1.30–2.87)**
		TT	79 (16.9)	18 (6.4)	**3.61 (2.08–6.27)**	**3.72 (1.91–7.25)**
		C	592 (63.2)	430 (76.5)	—	—
		T	344 (36.7)	132 (23.5)	**1.95 (1.53–2.47)**	
	rs1799930 (G590A)	GG (ref.)	386 (82.7)	222 (78.4)	—	—
		GA	72 (15.4)	60 (21.2)	0.69 (0.47–1.01)	0.79 (0.49–1.28)
		AA	9 (1.9)	1 (0.4)	5.18 (0.65–41.13)	8.72 (0.95–80.14)
		G	844 (90.4)	504 (89.0)	—	—
		A	90 (9.6)	62 (11.0)	0.86 (0.61–1.22)	
	rs1208 (A803G)	AA (ref.)	203 (43.6)	149 (53.2)	—	—
		AG	195 (41.8)	110 (39.3)	1.30 (0.95–1.78)	1.44 (0.97–2.14)
		GG	68 (14.6)	21 (7.5)	**2.38 (1.39–4.05)**	**3.25 (1.73–6.10)**
		A	601 (64.5)	408 (72.9)	—	—
		G	331 (35.4)	152 (27.1)	**1.47 (1.75–1.86)**	
	rs1799931 (G857A)	GG (ref.)	322 (69.2)	191 (68.5)	—	—
		GA	115 (24.7)	56 (20.1)	1.22 (0.84–1.76)	1.27 (0.81–1.98)
		AA	28 (6.0)	32 (11.5)	**0.52 (0.30–0.89)**	**0.34 (0.17–0.71)**
		G	759 (81.6)	438 (78.5)	—	—
		A	171 (18.4)	120 (21.5)	0.82 (0.63–1.06)	
NQO1^**^	rs1800566	CC (ref.)	154 (33.0)	78 (28.0)	—	—
		CT	224 (48.0)	145 (52.0)	0.78 (0.55–1.10)	0.80 (0.52–1.23)
		TT	89 (19.1)	56 (20.1)	0.80 (0.52–1.24)	0.91 (0.53–1.56)
		C	532 (57.0)	301 (54.0)	—	—
		T	402 (43.0)	257 (46.1)	0.98 (0.71–1.09)	
CYP2E1^**^	rs3813867	GG (ref.)	292 (64.3)	182 (66.2)	—	—
		GC	150 (33.0)	89 (32.4)	1.05 (0.76–1.45)	0.91 (0.60–1.38)
		CC	12 (2.6)	4 (1.5)	1.87 (0.59–5.88)	1.39 (0.34–5.70)
		G	734 (80.8)	453 (82.4)	—	—
		C	174 (19.2)	97 (17.6)	1.10 (0.84–1.45)	

OR, crude odds ratios; aOR, adjusted odds ratios by: child's sex, age, paternal education level, maternal education level, maternal age at pregnancy, Active smoking of the mother after birth, alcoholism of the mother before pregnancy, family cancer history, health institution, birthweight, Maternal exposure to hydrocarbons at home during pregnancy, x-rays exposure during pregnancy, alcoholism of the father before pregnancy, Active smoking of the father after birth. Genotyping rate

* >98% and

** *>96.6%. Bold values represent the most statistically significance results*.

In the genotype analysis, an association with ALL was observed, both under heterozygous and recessive models to the rs1801280 (T341C) (OR 1.44, 95% IC 1.04–1.98, and OR 2.25, 95% IC 1.33–3.80, respectively) and rs1789929 (C481T) (OR 1.59, 95% IC 1.16–2.20 and OR 3.61, 95% IC 2.08–6.27, respectively); as well, under recessive model for the rs1208 (A803G) (OR 2.38, 95% IC 1.39–4.05). *CYP2E1* (rs3813867) and *NQO1* (rs1800566) SNPs were not associated with ALL (Table 1).

No interactions among *NQO1* (rs1800566), *CYP2E1* (rs3813867), and *NAT2* (rs1799930, G590A) and the exposure variables were noted (Supplementary Table 3). Nevertheless, homozygotes genotypes TT and AA of *NAT2* SNPs rs1041983 (C282T) and rs1799931 (G857A), respectively, were associated with a reduced risk of ALL under exposure to hydrocarbons by the mother at home before and during pregnancy and after birth (HEMAB), drug consumption, active smoking, alcohol consumption and insecticide exposure by the mother before pregnancy, whilst being pregnant and while child feeding. As well as active smoking and alcohol drinking by father before and during pregnancy. *NAT2* variants rs1799929 and rs1208 variants, which were associated with ALL risk, exhibited an increased ORs under maternal hydrocarbons exposure and drug consumption. In the same way, active smoking, and alcohol consumption by the parents during pregnancy incremented the risk to ALL in homozygotes cases for mutant alleles to both SNPs. In fact, the rs1799929 (C481T) and active smoking by the father after birth displayed the highest OR (4.49, CI 95% 2.46–8.17) to ALL observed in the present analysis. The rs1799930 (*NAT2*), rs1800566 (*NQO1*), and rs3813867 (*CYP2E1*) were not associated neither after adjusting by these variables (Supplementary Table 3).

### Linkage Disequilibrium (LD) and Haplotypes Analyses

The assessment of LD displayed low LD among *NAT2* polymorphisms (Supplementary Figure 1). By including all *NAT2* variants, we found 11 haplotypes showing frequencies >0.01 as described in Table 2. Rapid alleles as NAT2^*^4, NAT2^*^11A, NAT2^*^12A, NAT2^*^12C, and NAT2^*^13A were identified. Slow alleles as NAT2^*^5B, NAT2^*^5C, NAT2^*^5V, NAT2^*^6A, NAT2^*^7A, and NAT2^*^7B, were detected. Statistically significant differences were noted for NAT2^*^4, NAT2^*^7B, and NAT2^*^13A distribution between cases and controls. Even though NAT2^*^5C, NAT2^*^11A, and NAT2^*^12C, also showed significant differences between cases and controls. Nonetheless, due to the CI values, the associations should be taking with caution (Table 2).

**Table 2:** Distribution of the most frequent *NAT2* alleles in health children and in patients with acute lymphoblastic leukemia.

**Haplotype**	**Alleles**	**Phenotype^**a**^**	**Cases (*n* = 929)**	**Controls (*n* = 568)**	**OR (95% CI)**	***P*-values**
			***n* (%)**	***n* (%)**		
CTCGAG	NAT2^*^4	Rapid	291 (31.3)	210 (36.9)	0.78 (0.62–0.97)	0.0263
CCTGGG	NAT2^*^5B	Slow	193 (20.8)	105 (18.6)	1.16 (0.88–1.52)	0.3054
TTCGAA	NAT2^*^7B	Slow	94 (10.1)	82 (14.5)	0.67 (0.48–0.93)	0.0109
TTCAAG	NAT2^*^6A	Slow	60 (6.4)	48 (8.5)	0.75 (0.49–1.13)	0.128
CTCGGG	NAT2^*^12A	Rapid	55 (5.9)	23 (4.0)	1.49 (0.88–2.53)	0.1075
TTCGAG	NAT2^*^13A	Rapid	17 (1.9)	23 (4.0)	0.44 (0.22–0.87)	0.0128
CTCGAA	NAT2^*^7A	Slow	16 (1.8)	19 (3.3)	0.51 (0.25–1.04)	0.0495
CCCGGG	NAT2^*^5C	Slow	28 (3.0)	6 (1.1)	2.91 (1.14–7.88)	0.017
CTTGAG	NAT2^*^11A	Rapid	28 (3.0)	1 (0.2)	17 (2.05–348.8)	0.00001^*^
CTTGGG	NAT2^*^12C	Rapid	19 (2.1)	2 (0.4)	5.91 (1.32–36.83)	0.0095^*^
TCTGAG	NAT2^*^5V	Slow	16 (1.7)	1 (0.2)	9.94 (1.39–201.58)	0.0097^*^

Haplotypes of NAT2 SNPs rs1041983 C/T, rs1801280 T/C, rs1799929 C/T, rs1799930G/A, rs1208A/G, and rs1799931G/A were obtained by HAPLOVIEW. The haplotype frequencies <0.01 are not displayed.

a Phenotype inferred base on the belonging group NAT2

* 5. OR, crude odds ratio; 95% CI, 95% Confidence interval.

*P-values remain under 10,000 permutation test. Bold values represent the most statistically significance results.

### Frequency of Rapid and Slow Acetylators

Patients harboring wild type for all of the *NAT2* polymorphisms were classified as rapid acetylators. Those with homozygous mutants or heterozygous for more than one of the polymorphisms were phenotyped as slow acetylators, being the remainder considered as intermediate acetylators (Table 3). Overall cases and controls, 458 and 269 phenotypes, respectively, were established. Rapid acetylator frequency was 20.7%, intermediate phenotype was observed in 45.9% and slow in 33.4% of the children with ALL. Meanwhile, 25.3% of the control population was comprised by rapid acetylators, and intermediate and slow were found in 39.8 and 34.9%, respectively. No statistically significant differences were found (Table 3).

**Table 3:** Association between acetylator phenotypes and risk of childhood acute lymphoblastic leukemia.

**Acetylator phenotype**	**Cases**	**Controls**	**OR (95% CI)**	**aOR (95% CI)**
	***n* = 469**	***n* = 285**		
	***n* (%)**	***n* (%)**		
Rapid (ref.)	95 (20.7)	68 (25.3)	—	—
Intermediate	210 (45.9)	107 (39.8)	1.40 (0.95–2.07)	1.83 (1.11–3.02)
Slow	153 (33.4)	94 (34.9)	1.16 (0.78–1.74)	1.21 (0.72–2.04)

*OR, crude odds ratios; aOR, adjusted odds ratios by: child's sex, age (≥6 years), maternal education level, maternal age at pregnancy, active smoking of the mother after birth, alcoholism of the mother before pregnancy, family cancer history, health Institution, birthweight, maternal exposure to hydrocarbons at home during pregnancy, x-rays exposure during pregnancy, alcoholism of the father before pregnancy, active smoking of the father after birth. Missing data for phenotype classification: 2 and 5.6% for cases and controls, respectively*.

### Gene-Gene Interaction Between NQ01-CYP2E1-NAT2 Polymorphisms

**Figure 1:**
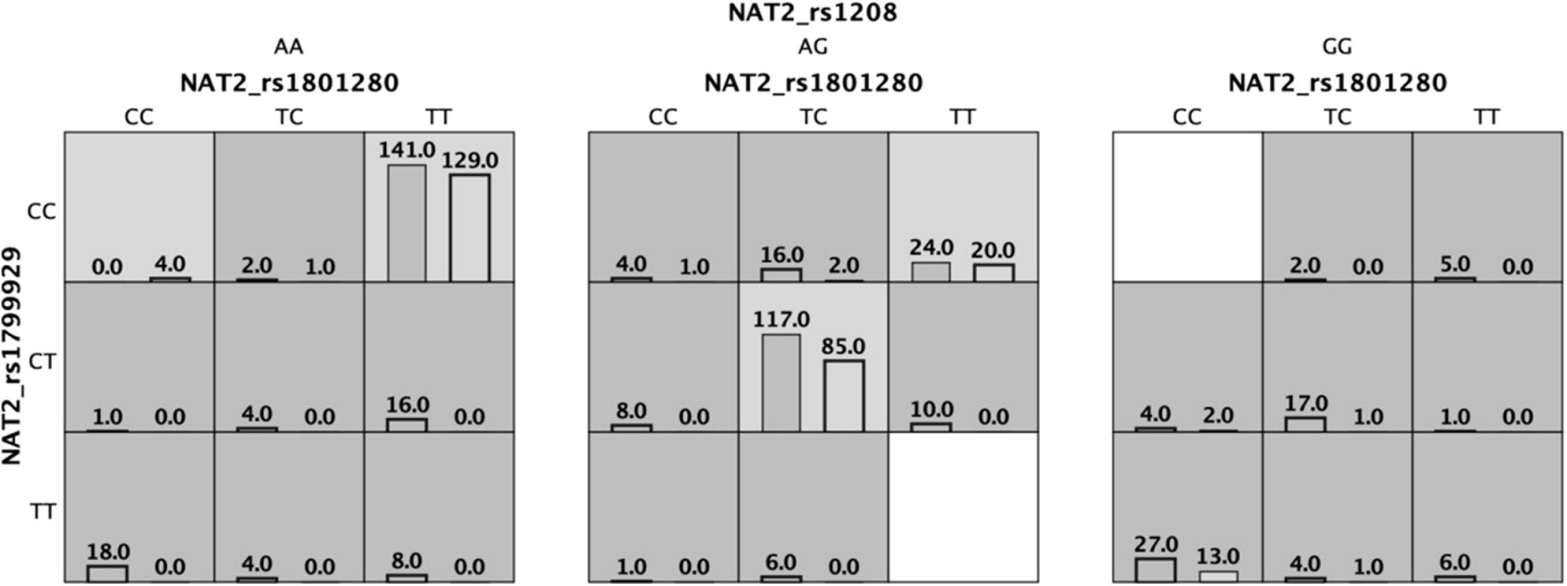
Three *locus* multifactor dimensionality reduction (MDR) model demonstrating the effect of *NAT2* on the association with the risk of developing acute lymphoblastic leukemia. Dark cells mean high risk and light cells are low risk. Cases are shown in the left bar and controls are shown in the right bar.

To investigate for interactions among *NQ01-CYP2E1*-*NAT2* genotypes, we performed a MDR analysis including cases and controls having complete genotyping data. The model with the lowest prediction error and highest cross-validation consistency (CVC) was selected (Table 4). The rs1799929 was the best factor model which showed statistically significant difference with testing accuracy (TBA) of 0.5856, a 10/10 CVC (Table 4) and contributing with 3.03% to the risk of ALL. The multilocus model with CVC (10/10) and minimum prediction error (maximum testing accuracy is 0.6377) was a three-factors model including the *NAT2*_rs1801280, rs1799929, and rs1208. These three *NAT2* SNPs interact together to collectively increase the risk to develop ALL (OR 6.59, CI 95% 4.05–10.71) (Figure 1, Table 4).

**Table 4:** Gene-gene interaction analysis between *NAT2, NQ01*, and *CYP2E1* polymorphisms and acute lymphoblastic leukemia risk.

**Number of factors**	**Model**	**TA**	**TBA**	**CVC**	**OR (CI 95%)**	***p*-value^**°**^**
1	***NAT2***-rs1799929*	**0.585**	**0.585**	**10/10**	**1.99 (1.46–2.31)**	<0.0001
2	*NAT2*-rs1801280, *NAT2*-rs1799929	0.614	0.6031	8/10	5.42 (3.26–9.02)	<0.0001
3	***NAT2*****-rs1801280**, ***NAT2*****-rs1799929**, ***NAT2***-rs1208**	**0.644**	**0.637**	**10/10**	**6.59 (4.05, 10.71)**	**<0.0001**
4	*NAT2*-rs1041983, *NAT2*-rs1801280, *NAT2*-rs1799929, *NAT2*-rs1799931	0.662	0.5909	6/10	3.75 (2.71–5.19)	<0.0001
5	*NAT2*-rs1041983, *NAT2*-rs1799929, *NAT2*-rs1799931, *NQ01*-rs1800566, *CYP2E1*-rs3813867	0.689	0.568	3/10	6.19 (4.18–9.15	<0.0001
6	*NAT2*-rs1041983, *NAT2*-rs1799929, *NAT2*-rs1208, *NAT2*-rs1799931, *NQ01*-rs1800566, *CYP2E1*-rs3813867	0.715	0.549	5/10	5.89 (4.21–8.26)	<0.0001
7	*NAT2*-rs1041983, *NAT2*-rs1799929, *NAT2*-rs1799930, NAT2-rs1208, *NAT2*-rs1799931, *NQ01*-rs1800566, *CYP2E1*-rs3813867	0.738	0.5751	4/10	7.33 (5.19–10.35)	<0.0001
8	*NAT2*-rs1041983, *NAT2*-rs1801280, *NAT2*-rs1799929, *NAT2*-rs1799930, *NAT2*-rs1208, *NAT2*-rs1799931, *NQ01*-rs1800566, *CYP2E1*-rs3813867	0.754	0.578	10/10	8.85 (6.23–12.59)	<0.0001

Best one-to-eight-locus model of the NAT2-NQ01-CY2E1 gene–gene interactions with cross-validation consistency (CVC) and prediction error per the n-locus model that have been obtained by the multifactor- dimensionality reduction (MDR) of our data set. TA, training balance accuracy; TBA, testing balance accuracy; *The best one factor model and **The best multilocus model. °P-values from whole statistics data set. Bold values indicate the best single* and models.

**Figure 2:**
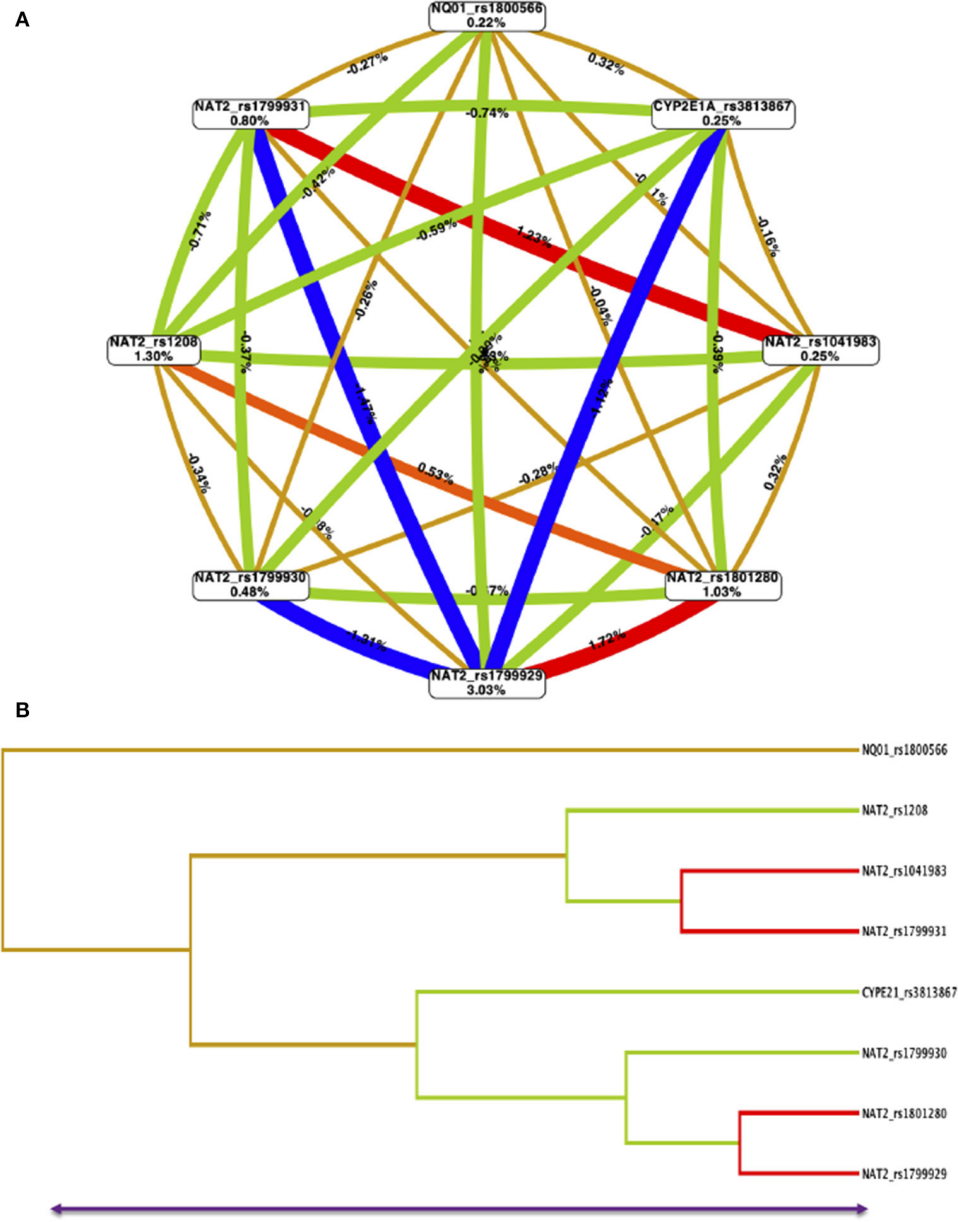
Multifactor dimensionality reduction (MDR) for NAT2-NQ01-CYP2E1A SNPs interactions model. **(A)** The interaction circle graph was built by SNPs (boxes) with pairwise connections among them. Values in each box show information gained by each SNP, and values and color between nodes are interaction effects. A synergistic or non-additive effect is represented by positive values and redundancy or correlation is indicated by negative values. Red lines mean strong synergistic interaction than blue lines and green lines indicates redundancy. The gold lines indicate independence. NAT2-rs1799929 has the larger effect on susceptibility to ALL (3.03%). Combining NAT2-rs1799929-rs1801280, as well, NAT2-rs10418983-rs1799931 provides more information about case-control status than considering them additively. **(B)** The dendrogram graphic shows the pattern and level of interaction between all evaluated SNPs.

The interaction entropy analysis showed that *NAT2*-rs1799929 had the larger effect on the susceptibility to develop ALL (3.0%). A synergistic interaction (read lines) between *NAT2*-rs104183-rs1799931 and *NAT2*-rs1799929-rs1801280 SNPs, which reveals an epistasis effect between them, was observed. Redundancy (green lines) was observed between several *NAT2* and *CYP2E1* SNPs which means that jointly those SNPs provide less information than studying one SNP. Gold lines showed independence among SNPs (Figure 2).

### Genetic Structure of Mexican Children With Acute Lymphoblastic Leukemia and Controls

**Figure 3:**
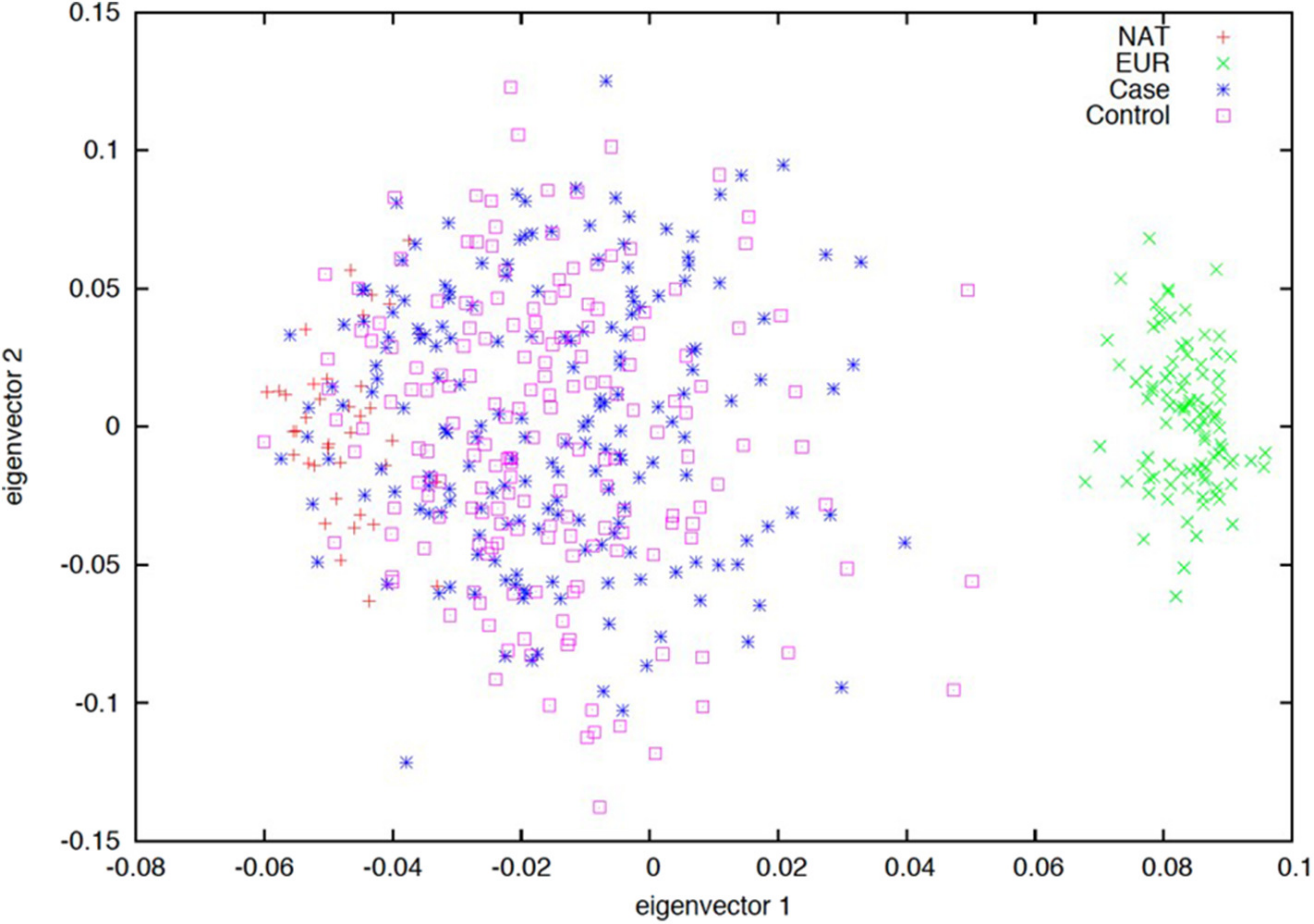
Ancestry estimation based on 32 ancestry informative markers (AIMs). Principal component analysis (PCA) plot of the first two eigenvectors. Spatial ancestry distribution of acute lymphoblastic leukemia (blue), controls (pink), European (green), and Native-American (red) populations. Dispersion of samples suggests a similar genetic background between cases and controls being enriched by a Native-American contribution.

The ancestry composition of the first subset of patients with ALL (*n* = 166) and controls (*n* = 167) who met the selection criteria during the study period was evaluated. A total of 32 AIMs, previously validated in a Mexican population were used (22). We found a similar genetic background among cases and controls, which were enriched by a Native-American (NA) contribution (Figure 3).

### Gene-Environment Interaction in Acute Lymphoblastic Leukemia

MDR was used to explore gene-environment interactions. The following maternal exposure to hydrocarbons in the pre-conceptional, during pregnancy and after birth periods, were considered in the analyses: global exposure to hydrocarbons, active and passive smoking, alcohol, drugs and medicine consumption, wood smoke, insecticides, benzene, gasoline, and petroleum exposures. Whilst environmental exposure factors assessed for the father were: active and passive smoking and alcohol dinking mainly for the pre-conceptional and during pregnancy periods.

For maternal exposure to hydrocarbons during preconceptional and after birth periods, the best combination was determined for hydrocarbons exposure, active and passive smoking, drugs and medicine consumption and alcohol, as well as insecticides, benzene, gasoline, petroleum, and wood smoke exposures; in addition to active and passive smoking and alcohol consumption by the father.

**Figure 4:**
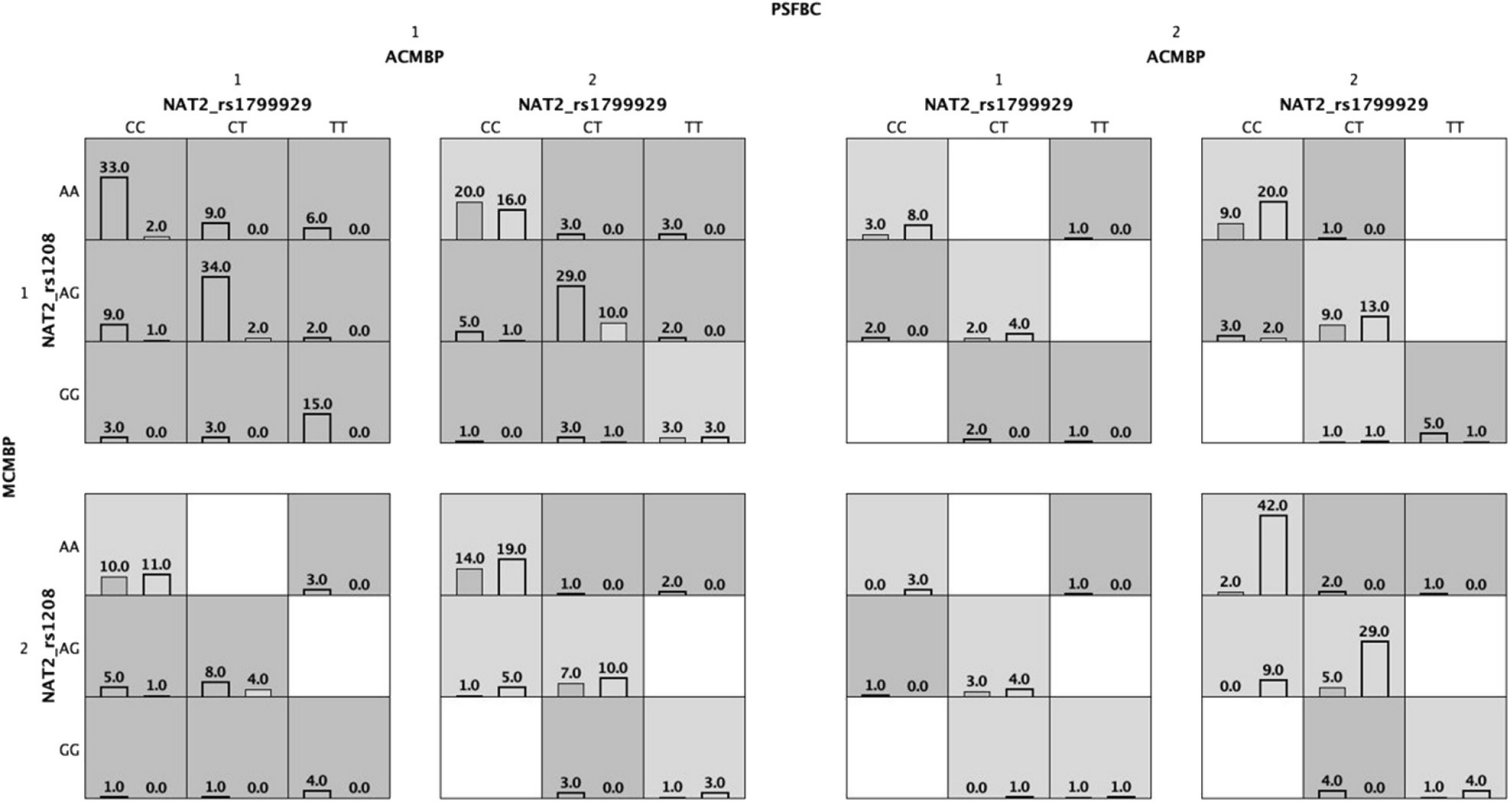
Multifactor dimensionality reduction (MDR) analysis of gene-environment interactions associated with the risk of acute lymphoblastic leukemia. For each cell, cases and controls are indicated on the left and right bars, respectively. PSFBC: passive smoking by the father before conception of index child. ACMBP: alcohol consumption by the mother before pregnancy on index child; 1: positive, 2: negative.

Our analysis suggests that passive smoking by the father before conception (PSFBC) and during pregnancy (PSFDP) was the best factor with statistical significance (TBA 0.7087 CVC 10/10 and TBA 0.6868 CVC 10/10, respectively). The multifactor model with a minimum prediction error (TBA 0.7648) and CVC (10/10) was *NAT2*_rs1799929-*NQ01*_rs2811566-*CYP2E1*_rs1803867-alcohol consumption by the mother before pregnancy (ACMBP)-medicine consumption by the mother before pregnancy (MCMBP)-PSFBC suggesting that these factors jointly contributed to the etiology of ALL (Figure 4, Table 5).

**Table 5:** Gene-environment interaction and acute lymphoblastic leukemia risk prediction.

**Exposure Window**	**# Factors**	**Model**	**TA**	**TBA**	**CVC**	**OR (CI 95%)**	*P*-values*
Before pregnancy	1	PSFBC	0.7087	0.7087	10/10	6.48 (1.90–22.08)	<0.0001
	2	MCMBP, PSFBC	0.7245	0.6962	8/10	8.19 (5.36–12.50)	<0.0001
	3	NAT2_rs1799929, MCMBP, PSFBC	0.7466	0.7061	5/10	8.58 (5.78–12.74)	<0.0001
	4	NAT2_rs1799929, NAT2_rs1208, MCMBP, PSFBC	0.773	0.7339	8/10	11.70 (7.72–17.72)	<0.0001
	5	NAT2_rs1799929, NAT2_rs1208, ACMBP, MCMBP, PSFBC	0.7965	0.7648	10/10	18.98 (11.72–30.74)	<0.0001
	6	NAT2_rs1799929, NQ12_rs2811566, CYP2E1A_rs3813867, ACMBP, MCMBP, PSFBC	0.8249	0.6658	3/10	19.04 (12.28–29.52)	<0.0001
	7	NAT2_rs1042983, NAT2_rs1208, NQ12_rs1800566, MEHBP, ACMBP, MCMBP, PSFBC	0.8689	0.6145	5/10	39.31 (23.74–65.12)	<0.0001
	8	NAT2_rs1041983, NAT2_rs1208, NQ01_rs1800566, MEHBP, ASMBP, ACMBP, MCMBP, ASFBC, PSFBC	0.9363	0.5382	2/10	70.72 (40.38–123.86)	<0.0001
During Pregnancy	1	PSFDP	0.6889	0.6868	10/10	6.68 (4.43–10.07)	<0.0001
	2	ASMP, PSFDP	0.6987	0.6784	7/10	7.51 (4.94–11.42)	<0.0001
	3	NAT2_rs1799929, PSMP, PSFDP	0.7141	0.6603	5/10	8.05 (5.34–12.16)	<0.0001
	4	NAT2_rs1041983, NAT2_rs1799929, NQ01_rs1800566, PSFDP	0.7318	0.6491	4/10	7.15 (4.90–10.45)	<0.0001
	5	NAT2_rs1041983, NAT2_rs1799929, NQ01_rs1800566, CYP2E1A_rs3813867, PSFDP	0.7602	0.6352	6/10	10.19 (6.83–15.22)	<0.0001
	6	NAT2_rs1041983, NAT2_rs1799929, NAT2_rs1799931, NQ01_rs1041566, CYP2E1A_rs3813867, PSFDP	0.7945	0.6296	7/10	14.90 (9.80–22.65)	<0.0001
	7	NAT2_rs1041983, NAT2_rs1799929, NAT2_rs1799931, NQ01_rs1041566, CYP2E1A_rs3813867, HEMHDP, PSFDP	0.8295	0.6196	5/10	22.93 (14.68–35.80)	<0.0001
	8	NAT2_rs1041983, NAT2_rs1799929, NAT2_rs1799931, NQ01_rs1041566, CYP2E1A_rs3813867, HEMHDP, ASFDP, PSFDP	0.8608	0.5774	4/10	35.79 (22.15–57.85)	<0.0001
After birth	1	NAT2_rs1799929	0.6021	0.6021	10/10	2.29 (1.62–3.22	<0.0001
	2	NAT2_rs1799929, ASMAB	0.6286	0.6065	8/10	2.91 (2.04–4.14)	<0.0001
	3	NAT2_rs1041983, NAT2_rs1799929, NAT2_rs1799931	0.6561	0.606	6/10	3.52 (2.48–5.01)	<0.0001
	4	NAT2_rs1041983, NAT2_rs1799929, NAT2_rs1799931, NQ01_rrs1800566	0.6848	0.5899	5/10	4.54 (3.17–6.51)	<0.0001
	5	NAT2_rs1041983, NAT2_rs1799929, NAT2_rs1799931, NQ01_rrs1800566, IEAB	0.7109	0.5724	5/10	5.66 (3.92–8.17)	<0.0001
	6	NAT2_rs1041983, NAT2_rs1799929, NAT2_rs1799931, NQ01_rrs1800566, IEAB, ASFAB	0.7553	0.6062	9/10	9.22 (6.24–13.61)	<0.0001

Best models gene–environment interactions with cross-validation consistency (CVC) and prediction error per the n-locus model that have been obtained by the multifactor-dimensionality reduction (MDR) of our data set. Table is displaying best models with TBA > 0.57, *Values from Whole Statistics data set. TA, training balance accuracy; TBA, testing balance accuracy; PSFBC, passive smoking for the father before conception; MCMBP, Medicine consumption for the mother before pregnancy; ACMBP, alcohol consumption by the mother before pregnancy; MEHBP, hydrocarbons exposure by the mother before pregnancy; ASMP, active smoking for the mother during pregnancy; ASMBP, active smoking by the mother before pregnancy; PSFDP, passive smoking by the father during pregnancy; ASFP, active smoking by the father during pregnancy; HEMDP, hydrocarbon exposure by the mother during pregnancy; ASMAB, active smoking by the mother after child's birth; IEAB, insecticide exposure of the mother after birth; ASFAB, active smoking by the father after birth.

After child's birth, the MDR identified to *NAT2*_rs1799929 as the best one factor (TBA 6021 CVC 10/10) and the six-factor interaction model *NAT2*_rs1041983, *NAT2*_rs1799929, NAT2_rs1799931, *NQ01*_rs1800566-insecticide exposition after child's birth (IEAB)-active smoking by the father after child's birth (ASFAB) as the best model for ALL with a TBA of 0.6062 and CVC 9/10 (Table 5).

The interaction entropy analysis showed that PSFBC (13.39%), ACMBP (9.88), and MCMBP (9.65%) have the larger effect on susceptibility to ALL before pregnancy (Supplementary Figure 2), whilst the *NAT2*-rs1799929 SNP was during pregnancy (4.15%) and after child's birth (4.21%) (Supplementary Figures 2, 3).

## Discussion

Acute lymphoblastic leukemia, the most prevalent cancer in children, like other hematologic malignancies is likely to develop from a complex interaction between genetic and environmental factors. Hydrocarbons, such as benzene, pesticides, and air pollutants, are among the common xenobiotics that have been implicated in ALL etiology (13, 15). Detoxification enzymes play an important role to metabolize environmental carcinogens and it is well-known that polymorphisms in genes encoding for these enzymes may explain inter-individual differences in leukemia risk (16). To gain more knowledge on how these factors could be interplaying to influence disease risk and outcomes in ALL, and to provide a global picture of the genetic ancestry and admixture patterns of Mexican pediatric patients with ALL from central Mexico we conducted this study.

The present report focused on the effects of genetic variation in *NQO1, CYP2E1A*, and *NAT2* polymorphisms and their interaction with environmental factors, such as home exposure to fertilizers, insecticides, hydrocarbon derivatives (benzene, petroleum products, etc.), and parental tobacco smoking and wood smoking, among other variables in the risk of developing childhood ALL. In the present study, we found that *NAT2* polymorphisms, but not *NQO1* and *CYP2E1* polymorphisms were associated with ALL risk. *NQ01* modifies internal exposure to bioactivated carcinogens. NQ01 has been previously described as an anticancer enzyme. There is a documented a relation between *NQ01* (rs1800566) with several types of cancer, including infant leukemia (31–35); however, its association with ALL is controversial. Our results are in contrast with findings of some studies (34, 35), but they are consistent with other reports that did not show a significant association between *NQO1* rs1800566 polymorphism and the risk of childhood ALL, when specific ethnic populations were analyzed (32, 36). Concerning *CYP2E1*, a fundamental contributor of the metabolisms of low molecular weight compounds as ethanol, and a bioactivator of many procarcinogens including benzene, has been associated with an increased risk of ALL (26). A differential contribution by the variants among populations could be influenced by age and ethnicity (26, 35, 36). We have only studied one SNP for each gene so far. Since a number of functional polymorphisms of *NQ01* and *CYP2E1* have been identified, we cannot discard the contribution of these genes in the risk of ALL in Mexican children.

*NAT2* is highly polymorphic and its activity is largely determined by coding single SNPs. To date, about 108 *NAT2* alleles have been identified by the Gene Nomenclature Committee (37), which defines three metabolizer groups: slow, intermediate and rapid acetylators. The wild-type NAT2^*^4 allele encodes a protein with high *N*-acetylation activity conferring a rapid acetylator phenotype (37, 38). Haplotypes containing more than one mutant allele at rs1041983, rs1801280 (*T341C*), rs1799929 (*C481T*), rs1799930, rs1208 (*A803G*), and rs1799931 (*G857A*) defined the low acetylator phenotype (29, 37). Our data showed that rs1801280 (T341C), rs1799929 (C481T), rs1208 (A803G), and rs1799931 (G857A) polymorphisms confer higher risk of ALL in Mexican children, which increases in homozygote mutant carriers, compared with heterozygotes. Notably, these risks were even higher after adjusting by age, sex, and other environmental variables (Table 3).

NAT2^*^4, considered the most common allele involved in rapid acetylation, was frequent in our control group and statistically significant differences were observed between cases and controls. Consistent with our findings, NAT2^*^5B has been reported to be the most common slow acetylator haplotype in Caucasian populations (4, 38). Notwithstanding, after multiple comparison tests, the statistical significance remained only for NAT2^*^11A, NAT2^*^12C, and NAT2^*^5V alleles, which were less frequent in healthy children than in cases with leukemia. Although similar frequencies of these alleles in healthy subjects have been reported elsewhere (39, 40), we cannot discard that these results could be biased by the small sample size of the control group, which is suggested by the wide CI values observed (Table 4).

Previous studies have suggested that the rapid-acetylator genotype of NAT2 leads to an increased risk of various types of cancer, particularly leukemia, colorectal and bladder cancer (4, 41–45). As it has been reported in Caucasian and Middle Eastern populations, rapid acetylator phenotypes were less frequent than slow acetylator phenotypes (4, 39, 46), but no statistical significance was observed between cases and controls. The predicted haplotypes described as slow acetylator TCTGAG (NAT2^*^5V, CI 1.39–201.58) and rapid CTTGAG (NAT2^*^11A, CI 2.05–348.8) and CTTGGG (NAT2^*^12C, CI 1.32–36.83) haplotypes, which were rare in the healthy subjects (<1%), were associated with higher risk of ALL. These results seem paradoxical; however, we predicted the acetylation phenotype according to the results of the *NAT2* haplotypes and potential haplotype misclassification could exist (5, 40). Uncommon haplotypes could not be clearly determined via indirect method of computational haplotype inference, however, direct methods for genotyping *NAT2* haplotypes are costly and have large turnaround time. Recently, a new method has been described to eliminate potential errors in the genotypes assignation, but, this technique is based on the NAT2^*^4, ^*^5B, ^*^6A, ^*^7B, ^*^12A, and ^*^13A, which are the six most common NAT2 haplotypes in diverse populations. Due the differences in the *NAT2* SNPs and haplotype frequencies among the populations, this new method is no applicable in very heterogenous ethnic groups, such as Mexican. In addition, it has been reported a genotype-phenotype discordance (47, 48). It is well-known that the acetylation phenotype is affected either by *NAT2* genetic variants or by environmental variables.

It has been mentioned that diet and epigenetic factors could be potential modifiers of discordant association results reported by studies performed in ALL. Recent studies have suggested that dietary lifestyle has a significant influence on xenobiotic metabolism by modifying the gut microbiota and consequently *NAT2* gene expression in the liver. Subjects from the same ethnic group but living in different geographic regions can be capable of responding differently to xenobiotic agents (49). Moreover, inconsistent and even contradictory data is not surprising, since multiple genes have been implicated in key pathways associated with leukemogenic processes. More over, studies of two or three genotypes in combination have also yielded inconsistent results.

It has also been hypothesized that slow acetylator phenotypes have suffered positive selection in populations under an insufficient folate diet (50). Studies in Mexican children have documented a folate deficiency in 11.2% of the children aged 4 years, which could explain our results (51). Nevertheless, our findings in the control group differ of those frequencies reported in general population from different regions of Mexico (43, 50, 52). *NAT2* gene has a high frequency of functional variation that differs among ethnically diverse populations, in fact, *NAT2* functional variation contributes to high levels of diversity, illustrating how geographically and temporally fluctuating xenobiotic environments may have influenced our genome variability and susceptibility to disease (52, 53). We discarded population stratification because we performed an ancestry structure analysis in a subset of patient and controls randomly selected. Our data showed that ALL cases, as well as control subjects belong to Mestizo group, the main ethnic group of Mexican population. The ancestry composition observed in the present study is in accordance with the Conquest history of Mexican population, mostly comprised by European- and Amerindian-descendent groups (54, 55).

Association analysis stratifying for exposure variables revealed that the homozygotes to the risk allele of rs1041983 (C282T) and rs1799931 (G857A) confer protection to ALL under parents exposition to diverse xenobiotics (mother: hydrocarbons, drug consumption, active smoking, alcohol consumption and insecticide; father: active smoking and alcohol drinking). To our knowledge, no previous studies have explored the relationship between *NAT2* genotypes and these environmental factors in ALL. However, differential contribution of single NAT2 SNPs to the risk of ALL has been observed recently by Zhu et al., who performed a meta-analysis including 1,522 acute leukemia patients and 2,688 controls (11).

Since ALL is considered a multifactorial disease where xenobiotics could be important factors that contribute to its pathogenesis, and that *NQO1, CYP2E1*, and *NAT2* are enzymes involved in the metabolism of xenobiotics (including benzene, cigarette smoking, chemotherapy agents, and alcohol drinking), which increase the risk to develop diverse human diseases (2, 11, 19), we used a MDR approach to identify combinations between genetic and environmental factors associated with ALL (29). Our data showed that the *NAT2*_rs1799929- *NAT2*_rs1208-ACMBP-MCMBP-PSFBC interact to increase the risk of ALL before pregnancy.

The *NAT2*_rs1041983, *NAT2*_rs1799929, NAT2_rs1799931, *NQ01*_rs1800566, IEAB, ASFAB was the best model after child's birth; meaning that these factors in combination increase the risk of ALL (Table 5). Our data suggest that the polymorphism affect acetylation of chemicals compounds having aromatic amines as drugs, pesticides, cigarette smoke, increase the risk to develop acute ALL in Mexican children. An interaction between *NAT2* and alcohol drinking and smoking with various outcomes have been reported. It was suggested that NAT2 could be involved in the activation of one or more pro-carcinogens associated with alcohol intake and the risk of oral squamous cell cancer (56). It is known that *NAT2* contributes to detoxification of tobacco smoke, pesticides and even prescription drugs (57–59); nonetheless, there are no evidences reporting a direct interaction within *NAT2* polymorphisms and the environmental factors identified in the present study with the risk to develop hematological diseases. The underlying mechanism for the link between genetic polymorphisms in these genes and insecticide in the development of ALL is not fully understood. A study conducted in infant leukemia with maternal exposure to dipyrone during pregnancy reported that *NAT2* SNPs are associated with this malignancy regardless of maternal exposure to the medication (59). Notwithstanding, it is well-known that studies addressing multi-gene rather than single-gene polymorphisms in xenobiotic genes could improve our knowledge of the genetic risk factors involved in ALL pathogenesis (4, 6, 25, 60).

This is a study that included patients from 8 public hospitals in Mexico City, what represents slightly <70% of all cases with leukemia in this city (61). It is also the first investigation to evaluate the interaction between *NQO1* (rs1800566), *CYP2E1* (rs3813867), and *NAT2* [rs1041983 (C282T), rs1801280 (T341C), rs1799929 (C481T), rs1799930 (G590A), rs1208 (A803G), and rs1799931 (G857A)] polymorphisms and exposure to common environmental hydrocarbons. In Supplementary Table 4, we can see the sociodemographic characteristics and the distribution of the hydrocarbon exposure variables of the children diagnosed with ALL of the Hospital that was not included in the present analysis. When comparing frequencies, there are no important differences to highlight, thus reducing the possibility of selection biases.

On the other hand, with respect to the variables included in this study to assess the exposure to hydrocarbons, we used a similar strategy reproduced in previous studies where the association between the exposure to hydrocarbons and the risk of developing childhood leukemia has been analyzed (62–65). This study had the disadvantage that it was limited to what the cases and controls remember, but our instrument included a question if the parents of the cases identified whether a possible cause of the disease was exposure to hydrocarbons, tobacco or alcohol consumption. There were no responses related to this. Thus, it can be noted that there is little likelihood of recall bias associated with the measurement of these variables. Another point in favor is that neither the interviewers nor the interviewees knew the results of the polymorphisms, so finding the existence of interactions between some variables and the polymorphisms reinforces the strategy with which our variables were measured. A logistic regression model was performed to evaluate the variables that could potentially be confusing, which is shown in Table 1. This was complemented with a recessive analysis which allowed us to adjust for possible confounding variables.

There were some limitations in the present study. On one hand, we tested only one SNP of *NQ01* and *CYP2E1* genes, thus we cannot discard the association among these genes and ALL or the interaction among SNPs in these genes and environmental xenobiotics. On the other hand, subjects were grouped into three different NAT2 acetylator phenotypes based only on six *NAT2* SNPs (slow acetylators: two slow alleles, intermediate acetylators: one slow and one rapid allele, and rapid acetylators: 2 rapid alleles). Considering that we did not study the rs1801279 (191G>A), the assessment of only these six SNPs could result in a misclassification of some *NAT2* alleles (39). In addition, new alleles of *NAT2* have been identified and there is there is phenotype heterogeneity within the slow and intermediate acetylator genotype groups, due to variation in enzyme activity conferred by different alleles (66). Low genotype rate could bias our phenotype results; nevertheless, it has been reported that using only the rs1041983 (C282T), rs1801280 (T341C) SNPs, it is possible to predict the NAT2 phenotype with high sensitivity and specificity (0.9993 and 0.9880, respectively) in Caucasian, Latin-American and Middle East populations (42, 67). Otherwise, controversial results in *NAT2* association findings among studies could be explained by factors, such as sample size, age group, genotyping method, and the time of exposure to risk agents (7, 68, 69).

## Conclusion

To the best of our knowledge, this is the first assessment of the interaction between hydrocarbon exposure and genetic polymorphisms of *NAT2, NQO1*, and C*YP2E1* on the risk of childhood ALL in the Mexican population, and the first report of ancestry background in Mexican children with ALL. Our study provides evidence that polymorphisms of *NAT2* might be genetic factors involved in childhood ALL. These results shed light on the contribution of *NAT2* polymorphisms to increase the risk of developing ALL in children.

## Data Availability Statement

The original contributions presented in the study are included in the article/Supplementary Material, further inquiries can be directed to the corresponding author/s.

## Ethics Statement

The studies involving human participants were reviewed and approved by the Ethics and National Committee of Scientific Research of the Instituto Mexicano del Seguro Social approved this study with number R-2013-785-062. Written informed consent to participate in this study was provided by the participants' legal guardian/next of kin.

## Author Contributions

AM-S, JM-A, and SJ-M: conceptualization. AM-S, JN-E, JCF-L, JM-A, and SJ-M: methodology. JN-E, JCF-L, JM-A, and SJ-M: formal analysis. AM-S, JN-E, and SJ-M: investigation. AM-S, EH-C, MP-S, AM-G, EJ-H, JM-T, HP-L, JF-L, RA-S, FM-R, JGP-G, DD-R, JT-N, JF-B, RE-E, PR-Z, LF-V, JG-U, SM-S, GE-A, CA-H, RR-C, LH-M, LG-L, GC-O, AG-E, IC-H, AM-H, ML-C, NH-P, JG-K, MR-V, DT-V, CC-R, FM-L, JAP-G, AM-R, AA-D, BS-D, VB-M, MM-R, OS-R, JR-B, HR-V, and AH-M: resources. AM-S and SJ-M: writing—original draft preparation. SJ-M and JM-A: writing—review and editing, supervision, and funding acquisition. All authors reviewed the final manuscript, read, and approved the submitted version.

## Conflict of Interest

The authors declare that the research was conducted in the absence of any commercial or financial relationships that could be construed as a potential conflict of interest.
