# Supplementary material for: Genotype-Environment Interaction Analysis of NQO1, CYP2E1, and NAT2 Polymorphisms and the Risk of Childhood Acute Lymphoblastic Leukemia: A Report From the Mexican Interinstitutional Group for the Identification of the Causes of Childhood Leukemia
Source: Front Oncol. 2020 Sep 21;10:571869. doi: 10.3389/fonc.2020.571869 (PMC7537417; doi:10.3389/fonc.2020.571869)
Supplement: Supplementary file 1 [file Table_1.docx]

| **Supplementary Table 1. General description of the study population** | | | | | | | |
| --- | --- | --- | --- | --- | --- | --- | --- |
| **Variables** | | **Subset of ALL cases*** | **Controls** | **OR (95% CI)** | **Total ALL cases** | **OR ( 95% CI)** | **p**** |
|  |  | **279** | **285** |  | **469** |  |  |
|  |  | ***n* ( %)** | ***n   (*%)** |  | ***n* ( %)** |  |  |
| Child´s sex | Male | 163 (58.4) | 157 (55.1) | 1.15 (0.82-1.60) | 277 (59.1) | 1.18 (0.87-1.58) | 0.28 |
|  | Female | 116 (41.6) | 128 (44.9) |  | 192 (40.9) |  |  |
| Child´s age at diagnosis / interview | 1-9.99 years | 173 (62.0) | 207 (72.6) | ------ | 286 (61.0) | ------ | **<0.001** |
|  | <1 years | 3 (1.1) | 10 (3.5) | 0.36 (0.10-1.32) | 3 (0.6) | 0.22 (0.06-0.80) |  |
|  | ≥ 10 years | 103 (36.9) | 68 (23.9) | 1.81 (1.26-2.62) | **180 (38.4)** | **1.92 (1.37-2.67)** |  |
| Weight at child´s birth | ≥ 3500g. | 81 (29.0) | 63 (22.1) | 1.44 (0.98-2.11) | 115 (24.5) | 1.14 (0.81-1.62) | 0.45 |
|  | <3500g. | 198 (71.0) | 222 (77.9) |  | 354 (75.5) |  |  |
| Family history of cancer | Yes | 114 (41.0) | 108 (37.9) | 1.14 (0.81-1.59) | 199 (43.2) | 1.24 (0.92-1.68) | 0.15 |
|  | No | 164 (59.0) | 177 (62.1) |  | 262 (56.8) |  |  |
| Maternal age at pregnancy | ≥35 years | 34 (12.2) | 34 (11.9) | 1.02 (0.62-1.70) | 54 (11.5) | 0.96 (0.61-1.52) | 0.85 |
|  | < 35 years | 245 (87.8) | 251 (88.1) |  | 416 (88.5) |  |  |
| Maternal education in years | 9.1-12.9 | 87 (31.2) | 120 (42.1) | ------- | 150 (32.0) | ------ | **0.02** |
|  | 0-9 | 147 (52.7) | 117 (41.1) | 1.73 (1.20-2.50) | **230 (49.0)** | **1.57 (1.13-2.18)** |  |
|  | ≥ 13 | 45 (16.1) | 48 (16.8) | 1.29 (0.79-2.11) | 89 (19.0) | 1.48 (0.97-2.27) |  |
| Paternal age at conception | ≥35 years | 88 (31.5) | 75 (26.3) | 1.29 (0.89-1.86) | 142 (30.3) | 1.22 (0.87-1.69) | 0.24 |
|  | < 35 years | 191(68.5) | 210 (73.7) |  | 327 (69.7) |  |  |
| Paternal education in years | 9.1-12.9 | 62 (22.2) | 105 (37.1) | ------ | 113 (24.1) | ------ | **<0.001** |
|  | 0-9 | 136 (48.7) | 139 (48.8) | 1.66 (1.12-2.45) | **214 (45.6)** | **1.43 (1.02-2.01)** |  |
|  | ≥ 13 | 81 (29.0) | 41 (14.4) | 3.35 (2.05-5.46) | **142 (30.3)** | **3.22 (2.08-4.98)** |  |
| Active smoking by the mother before conception | Yes | 61 (26.6) | 72 (25.3) | 1.07 (0.72-1.60) | 84 (22.5) | 0.86 (0.60-1.23) | 0.41 |
|  | No | 168 (73.4) | 213 (74.7) |  | 289 (77.5) |  |  |
| Active smoking by the mother during pregnancy | Yes | 3 (1.3) | 10 (3.5) | 0.36 (0.10-1.34) | 4 (1.1) | **0.30 (0.09-0.96)** | **0.03** |
|  | No | 226 (98.7) | 275 (96.5) |  | 369 (98.9) |  |  |
| Active smoking by the mother after birth | Yes | 30 (12.9) | 67 (23.5) | 0.48 (0.30-0.77) | **48 (12.6)** | **0.47 (0.31-0.70)** | <0.01 |
|  | No | 202 (87.1) | 218 (76.5) |  | 333 (87.4) |  |  |

| **Supplementary Table 1 (cont´). General description of the study population** | | | | | | | |
| --- | --- | --- | --- | --- | --- | --- | --- |
| **Variables** | | **Subset of ALL cases*** | **Controls** | **OR (95% CI)** | **Total ALL cases** | **OR ( 95% CI)** | **p**** |
|  |  | **279** | **285** |  | **469** |  |  |
|  |  | ***n* ( %)** | ***n   (*%)** |  | ***n* ( %)** |  |  |
| Alcohol consumption by the mother before pregnancy | Yes | 104 (45.4) | 233 (81.8) | 0.19 (0.12-0.27) | **170 (45.7)** | **0.19 (0.13-0.27)** | **<0.001** |
|  | No | 125 (54.6) | 52(18.2) |  | 202 (54.3) |  |  |
| Alcohol consumption by the mother during pregnancy | Yes | 5 (2.2) | 15 (5.3) | 0.40 (0.14-1.12) | **6 (1.6)** | **0.29 (0.11-0.77)** | **<0.01** |
|  | No | 224 (97.8) | 270 (94.7) |  | 366 (98.4) |  |  |
| Drug consumption by the mother before pregnancy | Yes | 4 (1.7) | 6 (2.1) | 0.81 (0.22-2.90) | 4 (1.0) | 0.49 (0.14-1.75) | 0.26 |
|  | No | 230 (98.3) | 279(97.9) |  | 380 (99.0) |  |  |
| Maternal diabetes | Yes | 5 (2.2) | 3 (1.1) | 2.10 (0.50-8.87) | 5 (1.3) | 1.28 (0.30-5.40) | 0.74 |
|  | No | 224 (97.8) | 282 (98.9) |  | 367 (98.7) |  |  |
| Active smoking by the father before conception | Yes | 101 (49.0) | 159 (57.8) | 0.70 (0.49-1.01) | **148 (44.0)** | **0.57 (0.42-0.79)** | **<0.001** |
|  | No | 105 (51.0) | 116 (42.2) |  | 188 (56.0) |  |  |
| Active smoking by the father during pregnancy | Yes | 89 (43.2) | 134 (48.7) | 0.80 (0.56-1.15) | **130 (38.8)** | **0.67 (0.48-0.92)** | **0.01** |
|  | No | 117 (56.8) | 141 (51.3) |  | 205 (61.2) |  |  |
| Active smoking by the father after birth | Yes | 97 (46.9) | 143 (53.6) | 0.76 (0.53-1.10) | **140 (40.8)** | **0.60 (0.43-0.83)** | **<0.01** |
|  | No | 110 (53.1) | 124 (46.4) |  | 203 (59.2) |  |  |
| Alcohol consumption by the father before pregnancy | Yes | 153 (75.4) | 241 (89.6) | 0.36 (0.21-0.59) | **253 (76.7)** | **0.38 (0.24-0.61)** | **<0.001** |
|  | No | 50 (24.6) | 28 (10.4) |  | 77 (23.3) |  |  |
| X-rays during pregnancy in the mother | Yes | 3 (1.3) | 5 (1.8) | 0.73 (0.17-3.09) | 4 (1.0) | 0.59 (0.16-2.23) | 0.43 |
|  | No | 230 (98.7) | 280 (98.2) |  | 378 (99.0) |  |  |
| Maternal exposure to hydrocarbons at home before pregnancy | Yes | 86 (37.6) | 154 (54.0) | 0.51 (0.36-0.73) | **133 (35.8)** | **0.47 (0.34-0.65)** | **<0.001** |
|  | No | 143 (62.4) | 131 (46.0) |  | 239 (64.2) |  |  |
| Maternal exposure to hydrocarbons at home during pregnancy | Yes | 68 (29.7) | 61 (21.4) | 1.55 (1.04-2.31) | **105 (28.2)** | **1.44 (1.00-2.07)** | 0.04 |
|  | No | 161 (70.3) | 224 (78.6) |  | 267 (71.8) |  |  |
| Maternal exposure to hydrocarbons at home after birth | Yes | 132 (57.1) | 180 (63.2) | 0.78 (0.55-1.11) | **190 (50.1)** | **0.59 (0.43-0.80)** | **<0.001** |
|  | No | 99 (42.9) | 105 (36.8) |  | 189 (49.9) |  |  |
| Maternal exposure to insecticides after birth | Yes | 97 (42.0) | 117 (41.1) | 1.04 (0.73-1.48) | 145 (38.3) | 0.89 (0.65-1.22) | 0.46 |
|  | No | 134 (58.0) | 168 (58.9) |  | 234 (61.7) |  |  |
| Health Institution | IMSS | 121 (43.4) | 158 (55.4) | 0.62 (0.44-0.86) | **196 (41.8)** | **0.58 (0.43-0.78)** | **<0.001** |
|  | Other | 158 (56.6) | 127 (44.6) |  | 273 (58.2) |  |  |
| * Frequency-matched with controls by child´s sex and age (±18 months). IMSS: The Instituto Mexicano del Seguro Social; OR are crude odds ratios  **** P-values (Chi-square Test) for the differences between Total ALL cases and Controls.** | | | | | | | |

| **Supplementary Table 2. Comparison of clinical, demographic characteristics and different types of exposure between total and the subset of ALL cases** | | | | |
| --- | --- | --- | --- | --- |
| **Variables** | | **Subset of ALL cases*** | **Total ALL cases** | **p***** |
|  |  | **279** | **469** |  |
|  |  | ***n* ( %)** | ***n* ( %)** |  |
| Child´s sex | Male | 163 (58.4) | 277 (59.1) | 0.86 |
|  | Female | 116 (41.6) | 192 (40.9) |  |
| Child´s age at diagnosis | 1-9.99 years | 173 (62.0) | 286 (61.0) | 0.73 |
|  | <1 years | 3 (1.1) | 3 (0.6) |  |
|  | ≥ 10 years | 103 (36.9) | 180 (38.4) |  |
| WHO 2008 Classification | B-lymphoblastic leukemia with t(9;22)(q34.1;q11.2);BCR-ABL1 | 7 (2.5) | 12 (2.6) | 0.44 |
|  | B-lymphoblastic leukemia with t(v;11q23.3);MLL(KMT2A) rearranged | 8 (2.9) | 10 (2.1) |  |
|  | B-lymphoblastic leukemia/lymphoma with t(12;21)(p13.2;q22.1); ETV6-RUNX1 | 26 (9.3) | 35 (7.5) |  |
|  | B-lymphoblastic leukemia/ with t(1;19)(q23;p13.3);TCF3-PBX1 | 23 (8.2) | 36 (7.7) |  |
|  | B-lypmphoblastic leukemia not otherwise specified (NOS)** | 191 (68.4) | 338 (72) |  |
|  | T-lymphoblastic leukemia | 24 (8.7) | 38 (8.1) |  |
| NCI risk classification | Standard | 100 (35.8) | 161(34.3) | 0.68 |
|  | High | 179 (64.2) | 308 (65.7) |  |
| Weight at child´s birth | ≥ 3500g. | 81 (29.0) | 115 (24.5) | 0.17 |
|  | <3500g. | 198 (71.0) | 354 (75.5) |  |
| Family history of cancer | Yes | 114 (41.0) | 199 (43.2) | 0.56 |
|  | No | 164 (59.0) | 262 (56.8) |  |
| Maternal age at pregnancy | ≥35 years | 34 (12.2) | 54 (11.5) | 0.77 |
|  | < 35 years | 245 (87.8) | 416 (88.5) |  |
| Maternal education in years | 9.1-12.9 | 87 (31.2) | 150 (32.0) | 0.69 |
|  | 0-9 | 147 (52.7) | 230 (49.0) |  |
|  | ≥ 13 | 45 (16.1) | 89 (19.0) |  |
| Paternal age at conception | ≥35 years | 88 (31.5) | 142 (30.3) | 0.72 |
|  | < 35 years | 191(68.5) | 327 (69.7) |  |
| Paternal education in years | 9.1-12.9 | 62 (22.2) | 113 (24.1) | 0.91 |
|  | 0-9 | 136 (48.7) | 214 (45.6) |  |
|  | ≥ 13 | 81 (29.0) | 142 (30.3) |  |
| Active smoking by the mother before conception | Yes | 61 (26.6) | 84 (22.5) | 0.25 |
|  | No | 168 (73.4) | 289 (77.5) |  |
| Active smoking by the mother during pregnancy | Yes | 3 (1.3) | 4 (1.1) | 0.79 |
|  | No | 226 (98.7) | 369 (98.9) |  |
| Active smoking by the mother after birth | Yes | 30 (12.9) | 48 (12.6) | 0.90 |
|  | No | 202 (87.1) | 333 (87.4) |  |

| Supplementary Table 2 (cont´d). Comparison of clinical, demographic characteristics and different types of exposure between total and the subset of ALL cases | | | | |
| --- | --- | --- | --- | --- |
| Variables | | Subset of ALL cases* | Total ALL cases | p*** |
|  |  | 279 | 469 |  |
|  |  | *n* ( %) | *n* ( %) |  |
| Alcohol consumption by the mother before pregnancy | Yes | 104 (45.4) | 170 (45.7) | 0.94 |
|  | No | 125 (54.6) | 202 (54.3) |  |
| Alcohol consumption by the mother during pregnancy | Yes | 5 (2.2) | 6 (1.6) | 0.61 |
|  | No | 224 (97.8) | 366 (98.4) |  |
| Drug consumption by the mother before pregnancy | Yes | 4 (1.7) | 4 (1.0) | 0.48 |
|  | No | 230 (98.3) | 380 (99.0) |  |
| Maternal diabetes | Yes | 5 (2.2) | 5 (1.3) | 0.43 |
|  | No | 224 (97.8) | 367 (98.7) |  |
| Active smoking by the father before conception | Yes | 101 (49.0) | 148 (44.0) | 0.26 |
|  | No | 105 (51.0) | 188 (56.0) |  |
| Active smoking by the father during pregnancy | Yes | 89 (43.2) | 130 (38.8) | 0.31 |
|  | No | 117 (56.8) | 205 (61.2) |  |
| Active smoking by the father after birth | Yes | 97 (46.9) | 140 (40.8) | 0.16 |
|  | No | 110 (53.1) | 203 (59.2) |  |
| Alcohol consumption by the father before pregnancy | Yes | 153 (75.4) | 253 (76.7) | 0.73 |
|  | No | 50 (24.6) | 77 (23.3) |  |
| X-rays during pregnancy in the mother | Yes | 3 (1.3) | 4 (1.0) | 0.78 |
|  | No | 230 (98.7) | 378 (99.0) |  |
| Maternal exposure at home before pregnancy | Yes | 86 (37.6) | 133 (35.8) | 0.65 |
|  | No | 143 (62.4) | 239 (64.2) |  |
| Maternal exposure at home during pregnancy | Yes | 68 (29.7) | 105 (28.2) | 0.70 |
|  | No | 161 (70.3) | 267 (71.8) |  |
| Maternal exposure at home after birth | Yes | 132 (57.1) | 190 (50.1) | 0.09 |
|  | No | 99 (42.9) | 189 (49.9) |  |
| Maternal exposure to insecticides after birth | Yes | 97 (42.0) | 145 (38.3) | 0.36 |
|  | No | 134 (58.0) | 234 (61.7) |  |
| Health Institution | IMSS | 121 (43.4) | 196 (41.8) | 0.67 |
|  | Other | 158 (56.6) | 273 (58.2) |  |
| * Frequency-matched with controls by child´s sex and age (±18 months). IMSS: The Instituto Mexicano del Seguro Social ** Ploidy was not routinely performed in participant hospitals during the study period. ***P-value from X^2^ test between total cases (n=469) and subset of ALL cases (n=279) | | | | |

| **Supplementary table 3. Association between genotypes and childhood acute lymphoblastic leukemia stratifying for each of the exposure variables** | | | | | | | | | | |
| --- | --- | --- | --- | --- | --- | --- | --- | --- | --- | --- |
| **Gene** | **SNP ID** | **Genotypes** | **OR (95% CI)** | **Hydrocarbons Maternal exposure** at home **before** **pregnancy** | **Hydrocarbons Maternal exposure** at home **during pregnancy** | **Hydrocarbons Maternal exposur**e at home **after birth** | **X-rays** **during pregnancy** in the mother | **Drug** consumption by the **mother** **before pregnancy** | Active **smoking** by the **mother** **before pregnancy** | Active **smoking** by the **mother** **during pregnancy** |
|  |  |  |  | **aOR (95%CI)** | | | | | | |
| ***NAT2*** | **rs1041983** | CC(*ref.*) | **-------** | **-------** | **-------** | **-------** | **-------** | **-------** | **-------** | **-------** |
|  |  | CT | 0.80 (0.58-1.10) | 0.82 (0.58-1.15) | 0.79(0.56-1.10) | 0.82 (0.58-1.14) | 0.84 (0.60-1.18) | 0.83 (0.59-1.15) | 0.79 (0.56-1.10) | 0.79 (0.56-1.10) |
|  |  | TT | 0.71 (0.43-1.14) | **0.51 (0.29-0.88)** | **0.53 (0.31-0.91)** | **0.53 0.31-0.90)** | **0.58 (0.34-0.99)** | **0.59 (0.35-0.99)** | **0.53 (0.31-0.91)** | **0.54 (0.31-0.92)** |
|  | **rs1801280** | TT *(ref.)* | **-------** | **-------** | **-------** | **-------** | **-------** | **-------** | **-------** | **-------** |
|  |  | **TC** | **1.44 (1.04-1.98)** | **1.61 (1.15-2.27)** | **1.49 (1.06-2.08)** | **1.53 (1.09-2.15)** | **1.46 (1.05-2.04)** | **1.46 (1.05-2.04)** | **1.52 (1.09-2.13)** | **1.50 (1.07-2.10)** |
|  |  | **CC** | **2.25 (1.33-3.80)** | **2.45 (1.41-4.24)** | **2.35 (1.37-4.04)** | **2.31 (1.34-3.96)** | **2.38 (1.39-4.08)** | **2.43 (1.42-4.18)** | **2.44 (1.42-4.19)** | **2.46 (1.43-4.22)** |
|  | **rs1799929** | CC (ref.) | **-------** | **-------** | **-------** | **-------** | **-------** | **-------** | **-------** | **-------** |
|  |  | **CT** | **1.59 (1.16-2.20)** | **1.86 (1.32-2.63)** | **1.74 (1.24-2.44)** | **1.79 (1.28-2.52)** | **1.69 (1.21-2.37)** | **1.69 (1.21-2.36)** | **1.77 (1.26-2.47)** | **1.76 (1.25-2.46)** |
|  |  | **TT** | **3.61 (2.08-6.27)** | **4.33 (2.43-7.73)** | **3.88 (2.20-6.84)** | **4.00 (2.26-7.07)** | **3.87 (2.20-6.81)** | **3.99 (2.26-7.04)** | **4.10 (2.32-7.22)** | **4.19 (2.36-7.42)** |
|  | **rs1799930** | GG *(ref.)* | **-------** | **-------** | **-------** | **-------** | **-------** | **-------** | **-------** | **-------** |
|  |  | GA | 0.69 (0.47-1.01) | 0.64 (0.42-0.96) | 0.64 (0.42-0.96) | 0.65 (0.44-0.98) | 0.66 (0.45-0.99) | 0.66 (0.44-0.99) | 0.62 (0.41-0.94) | 0.62 (0.41-0.94) |
|  |  | AA | 5.18 (0.65-41.13) | 3.05 (0.36-25.62) | 4.70 (0.56-39.36) | 3.73 (0.44-31.45) | 4.17 (0.50-34.92) | 4.13 (0.49-34.56) | 4.21 (0.50-35.28) | 4.15 (0.50-34.76) |
|  | **rs1208** | AA *(ref.)* | **-------** | **-------** | **-------** | **-------** | **-------** | **-------** | **-------** | **-------** |
|  |  | AG | 1.30 (0.95-1.78) | **1.43 (1.02-2.00)** | **1.41 (1.01-1.97)** | **1.46 (1.04-2.04)** | **1.38 (0.99-1.92)** | 1.38 (0.99-1.93) | **1.42 (1.02-1.98)** | **1.41 (1.08-1.96)** |
|  |  | **GG** | **2.38 (1.39-4.05)** | **3.01-1.73-5.23)** | **3.06 (1.77-5.28)** | **2.90 (1.68-5.01)** | **2.88 (1.67-4.96)** | **2.91 (1.68-5.01)** | **3.03 (1.76-5.23)** | **3.07 (1.77-5.32)** |
|  | **rs1799931** | GG *(ref.)* | **-------** | **-------** | **-------** | **-------** | **-------** | **-------** | **-------** | **-------** |
|  |  | GA | 1.22 (0.84-1.76) | 1.29 (0.88-1.89) | 1.27 (0.87-1.86) | 1.30 (0.89-1.90) | 1.32 (0.91-1.93) | 1.30 (0.89-1.89) | 1.26 (0.86-1.85) | 1.25 (0.85-1.83) |
|  |  | **AA** | **0.52 (0.30-0.89)** | **0.48 (0.27-0.88)** | **0.47 (0.26-0.84)** | **0.47 (0.26-0.84)** | **0.49 (0.27-0.87)** | **0.47 (0.26-0.85)** | **0.46 (0.25-0.83)** | **0.45 (0.25-0.82)** |
| ***NQO1*** | **rs1800566** | CC (ref.) | **-------** | **-------** | **-------** | **-------** | **-------** | **-------** | **-------** | **-------** |
|  |  | CT | 0.78 (0.55-1.10) | 0.79 (0.55-1.14) | 0.81 (0.56-1.17) | 0.75 (0.53-1.09) | 0.77 (0.54-1.10) | 0.77 (0.54-1.10) | 0.81 (0.56-1.16) | 0.80 (0.56-1.15) |
|  |  | TT | 0.80 (0.52-1.24) | 0.87 (0.55-1.38) | 0.91 (0.58-1.43) | 0.83 (0.53-1.31) | 0.83 (0.53-1.30) | 0.81 (0.52-1.27) | 0.90 (0.57-1.41) | 0.91 (0.58-1.42) |
| ***CYP2E1*** | **rs3813867** | GG (ref.) | **-------** | **-------** | **-------** | **-------** | **-------** | **-------** | **-------** | **-------** |
|  |  | GC | 1.05 (0.76-1.45) | 0.96 (0.68-1.36) | 0.98 (0.70-1.38) | 1.00 (0.71-1.40) | 1.01 (0.72-1.42) | 1.02 (0.73-1.42) | 1.00 (0.71-1.40) | 0.98 (0.70-1.37) |
|  |  | CC | 1.87 (0.59-5.88) | 1.89 (0.56-6.36) | 1.70 (0.51-1.27) | 1.91 (0.57-6.35) | 1.69 (0.51-5.57) | 1.67 (0.51-5.54) | 1.75 (0.53-5.78) | 1.68 (0.51-5.53) |
| **Bold numbers** indicate differences greater than 10% were observed between adjusted and crude ratios | | | | | | | | | | |
| OR= crude odds ratio; aOR= adjusted odds ratio for the study variable displayed in each column | | | | | | | | | | |

| **Supplementary Table 3 (cont´d). Association between genotypes and childhood acute lymphoblastic leukemia stratifying for each of the exposure variables** | | | | | | | | | | | |
| --- | --- | --- | --- | --- | --- | --- | --- | --- | --- | --- | --- |
| **Gene** | **SNP ID** | **Genotypes** | **cOR (95% CI)** | Active **smoking** by the **mother** **after birth** | **Alcohol** consumption by the **mother before pregnancy** | **Alcohol** consumption by the **mother during pregnancy** | **Maternal** exposure to **insecticides after birth** | Active **smoking** by the f**ather before conception** | Active **smoking** by the **father during pregnancy** | Active **smoking** by the **father after birth** | **Alcohol** consumption by the **father** **before conception** |
|  |  |  |  | **aOR (95% CI)** | | | | | | | |
| **NAT2** | **rs1041983** | CC(*ref.*) | **-------** | **-------** | **-------** | **-------** | **-------** | **-------** | **-------** | **-------** | **-------** |
|  |  | CT | 0.80 (0.58-1.10) | 0.80 (0.57-1.12) | 0.88 (0.61-1.26) | 0.79 (0.57-1.11) | 0.84 (0.60-1.18) | 0.77 (0.54-1.10) | 0.75 (0.53-1.06) | 0.80 (0.56-1.13) | 0.76 (0.53-1.08) |
|  |  | TT | 0.71 (0.43-1.14) | **0.57 (0.34-0.97)** | **0.52 (0.29-0.94)** | **0.51 (0.30-0.88)** | **0.56 (0.33-0.95)** | 0.60 (0.34-1.04) | 0.60 (0.34-1.03) | 0.67 (0.39-1.15) | 0.56 (0.32-0.99) |
|  | **rs1801280** | TT *(ref.)* | **-------** | **-------** | **-------** | **-------** | **-------** | **-------** | **-------** | **-------** | **-------** |
|  |  | **TC** | **1.44 (1.04-1.98)** | **1.48 (1.06-2.08)** | **1.61 (1.13-2.31)** | **1.53 (1.09-2.14)** | **1.49 (1.06-2.08)** | **1.65 (1.16-2.35)** | **1.61 (1.13-2.28)** | **1.65 (1.16-2.35)** | **1.70 (1.19-2.43)** |
|  |  | **CC** | **2.25 (1.33-3.80)** | **2.43 (1.41-4.18)** | **2.03 (1.14-3.61)** | **2.40 (1.39-4.13)** | **2.40 (1.40-4.11)** | **2.66 (1.51-4.66)** | **2.64 (1.51-4.63)** | **2.50 (1.43-4.38)** | **2.58 (1.46-4.55)** |
|  | **rs1799929** | CC (ref.) | **-------** | **-------** | **-------** | **-------** | **-------** | **-------** | **-------** | **-------** | **-------** |
|  |  | **CT** | **1.59 (1.16-2.20)** | **1.70 (1.21-2.38)** | **1.85 (1.29-2.66)** | **1.78 (1.27-2.49)** | **1.73 (1.24-2.42)** | **1.91 (1.34-2.72)** | **1.90 (1.34-2.70)** | **1.87 (1.32-2.67)** | **1.89 (1.32-2.70)** |
|  |  | **TT** | **3.61 (2.08-6.27)** | **4.11 (2.32-7.26)** | **3.49 (1.91-6.36)** | **4.07 (2.30-7.22)** | **3.91 (2.22-6.88)** | **4.45 (2.46-8.05)** | **4.47 (2.47-8.08)** | **4.49 (2.46-8.17)** | **4.05 (2.23-7.34)** |
|  | **rs1799930** | GG *(ref.)* | **-------** | **-------** | **-------** | **-------** | **-------** | **-------** | **-------** | **-------** | **-------** |
|  |  | GA | 0.69 (0.47-1.01) | 0.63 (0.42-0.95) | 0.69 (0.45-1.08) | 0.62 (0.41-0.94) | 0.65 (0.44-0.98) | 0.66 (0.43-1.00) | 0.63 (0.42-0.96) | 0.68 (0.44-1.03) | 0.70 (0.46-1.07) |
|  |  | AA | 5.18 (0.65-41.13) | 4.82 (0.58-39.91) | 5.78 (0.66-50.32) | 5.39 (0.61-47.99) | 4.10 (0.49-34.39) | 3.79 (0.45-31.98) | 4.07 (0.48-34.27) | 4.06 (0.49-33.41) | 4.38 (0.51-37.29) |
|  | **rs1208** | AA *(ref.)* | **-------** | **-------** | **-------** | **-------** | **-------** | **-------** | **-------** | **-------** | **-------** |
|  |  | AG | 1.30 (0.95-1.78) | **1.39 (0.99-1.94)** | **1.43 (1.00-2.05)** | **1.41 (1.02-2.00)** | **1.41 (1.01-1.96)** | **1.42 (1.01-2.02)** | **1.41 (1.00-1.99)** | **1.41 (0.99-1.99)** | **1.48 (1.04-2.11)** |
|  |  | **GG** | **2.38 (1.39-4.05)** | **2.92 (1.68-5.05)** | **2.89 (1.62-5.14)** | **3.08 (1.78-5.34)** | **2.90 (1.68-4.99)** | **3.17 (1.80-5.57)** | **3.12 (1.78-5.48)** | **3.06 (1.72-5.43)** | **2.98 (1.69-5.27)** |
|  | **rs1799931** | GG *(ref.)* | **-------** | **-------** | **-------** | **-------** | **-------** | **-------** | **-------** | **-------** | **-------** |
|  |  | GA | 1.22 (0.84-1.76) | 1.25 (0.85-1.83) | 1.40(0.93-2.11) | 1.25 (0.85-1.83) | 1.32 (0.90-1.93) | 1.25 (0.84-1.86) | 1.25 (0.84-1.85) | 1.34 (0.90-1.99) | 1.18 (0.79-1.76) |
|  |  | **AA** | **0.52 (0.30-0.89)** | **0.43 (0.24-0.78)** | **0.36 (0.19-0.70)** | **0.45 (0.25-0.81)** | **0.48 (0.27-0.87)** | **0.48 (0.25-0.90)** | **0.46 (0.25-0.87)** | **0.46 (0.25-0.86)** | **0.41 (0.22-0.77)** |
| ***NQO1*** | **rs1800566** | CC (ref.) | **-------** | **-------** | **-------** | **-------** | **-------** | **-------** | **-------** | **-------** | **-------** |
|  |  | CT | 0.78 (0.55-1.10) | 0.76 (0.52-1.09) | 0.81 (0.55-1.19) | 0.80 (0.55-1.14) | 0.76 (0.53-1.09) | 0.80 (0.55-1.17) | 0.81 (0.56-1.18) | 0.78 (0.53-1.13) | 0.79 (0.54-1.16) |
|  |  | TT | 0.80 (0.52-1.24) | 0.85 (0.54-1.34) | 0.97 (0.60-1.58) | 0.89 (0.57-1.39) | 0.84 (0.53-1.31) | 0.89 (0.55-1.42) | 0.89 (0.55-1.42) | 0.87 (0.55-1.40) | 0.85 (0.53-1.38) |
| ***CYP2E1*** | **rs3813867** | GG (ref.) | **-------** | **-------** | **-------** | **-------** | **-------** | **-------** | **-------** | **-------** | **-------** |
|  |  | GC | 1.05 (0.76-1.45) | 1.00 (0.1-1.40) | 0.96 (0.66-1.37) | 0.99 (0.70-1.39) | 1.01 (0.72-1.41) | 0.95 (0.66-1.35) | 0.95 (0.66-1.35) | 1.00 (0.70-1.43) | 0.93 (0.65-1.34) |
|  |  | CC | 1.87 (0.59-5.88) | 1.80 (0.54-6.02) | 1.62 (0.45-5.79) | 1.86 (0.55-6.24) | 1.74 (0.53-5.77) | 1.21 (0.33-4.40) | 1.25 (0.34-4.51) | 1.19 (0.33-4.32) | 1.07 (0.29-3.96) |
| **Bold numbers** indicate differences greater than 10% were observed between adjusted and crude ratios | | | | | | | | | | | |
| OR= crude odds ratio; aOR= adjusted odds ratio for the study variable in displayed in each column | | | | | | | | | | | |

| **Supplementary Table 4. Comparison of sociodemographic features and hydrocarbons exposure variables between included and not included patients** | | | | |
| --- | --- | --- | --- | --- |
| **Variables** | | **Total ALL cases included** | **ALL cases**  **not included** | **OR (95% CI)** |
|  |  | **469** | **90** |  |
|  |  | ***n* (%)** | ***n* (%)** |  |
| Child´s sex | Male | 277 (59.1) | 47 (52.2) | 1.32 (0.84-2.07) |
|  | Female | 192 (40.9) | 43 (47.8) | -------- |
| Child´s age at diagnosis / interview | 1-9.99 years | 286 (61.0) | 60 (66.7) | -------- |
|  | <1 years | 3 (0.6) | 2 (2.2) | 0.31 (0.04-1.71) |
|  | ≥ 10 years | 180 (38.4) | 28 (31.1) | 1.35 (0.85-2.23) |
| Weight at child´s birth | ≥ 3500g. | 115 (24.5) | 31 (34.5) | 0.61 (0.38-1.01) |
|  | <3500g. | 354 (75.5) | 59 (65.5) | -------- |
| Family history of cancer | Yes | 199 (43.2) | 38 (41.6) | 1.04 (0.66-1.64) |
|  | No | 262 (56.8) | 52 (58.4) | -------- |
| Maternal age at pregnancy | ≥35 years | 54 (11.5) | 13 (15.0) | 0.77 (0.40-1.47) |
|  | < 35 years | 416 (88.5) | 77 (85.0) | -------- |
| Maternal education in years | 9.1-12.9 | 150 (32.0) | 18 (20.0) | -------- |
|  | 0-9 | 230 (49.0) | 52 (58.1) | 0.70 (0.44-1.11) |
|  | ≥ 13 | 89 (19.0) | 20 (21.9) | 0.81 (0.47-1.42) |
| Paternal age at conception | ≥35 years | 142 (30.3) | 25 (28.2) | 1.12 (0.68-1.86) |
|  | < 35 years | 327 (69.7) | 65 (71.8) | -------- |
| Paternal education in years | 9.1-12.9 | 113 (24.1) | 58 (64.3) | -------- |
|  | 0-9 | 214 (45.6) | 15 (16.7) | 4.18 (2.34-7.51) |
|  | ≥ 13 | 142 (30.3) | 17 (19.0) | 1.86 (1.06-3.27) |
| Active smoking by the mother before conception | Yes | 84 (22.5) | 15 (16.7) | 1.45 (0.79-2.66) |
|  | No | 289 (77.5) | 75 (83.3) | -------- |
| Active smoking by the mother during pregnancy | Yes | 4 (1.1) | 1 (1.1) | 0.96 (0.10-8.73) |
|  | No | 369 (98.9) | 89 (99.9) | -------- |
| Active smoking by the mother after birth | Yes | 48 (12.6) | 15 (16.6) | 0.72 (0.38-1.35) |
|  | No | 333 (87.4) | 75 (83.4) | -------- |

| **Supplementary Table 4 (cont`d). Comparison of sociodemographic features and hydrocarbons exposure variables between included and not included patients** | | | | |
| --- | --- | --- | --- | --- |
| **Variables** | | **Total ALL cases** | **Not included** | **OR (95% CI)** |
|  |  | **469** | **90** |  |
|  |  | ***n* ( %)** | ***n   (*%)** |  |
| Alcohol consumption by the mother before pregnancy | Yes | 170 (45.7) | 36 (40.5) | 1.26 (0.79-2.02) |
|  | No | 202 (54.3) | 54 (59.5) | -------- |
| Alcohol consumption by the mother during pregnancy | Yes | 6 (1.6) | 1 (1.1) | 1.45 (0.17-12.2) |
|  | No | 366 (98.4) | 89 (99.9) | -------- |
| Drug consumption by the mother before pregnancy | Yes | 4 (1.0) | 1 (1.1) | 0.93 (0.10-8.48) |
|  | No | 380 (99.0) | 89 (99.9) | -------- |
| Maternal diabetes | Yes | 5 (1.3) | 1 (1.1) | 1.21 (0.14-10.51) |
|  | No | 367 (98.7) | 89 (99.9) | -------- |
| Active smoking by the father before conception | Yes | 148 (44.0) | 44 (56.1) | 1.06 (0.68-1.67) |
|  | No | 188 (56.0) | 56 (43.9) | -------- |
| Active smoking by the father during pregnancy | Yes | 130 (38.8) | 43 (47.7) | 0.69 (0.43-1.10) |
|  | No | 205 (61.2) | 47 (52.3) | -------- |
| Active smoking by the father after birth | Yes | 140 (40.8) | 42 (46.3) | 0.78 (0.49-1.25) |
|  | No | 203 (59.2) | 48 (53.7) | -------- |
| Alcohol consumption by the father before pregnancy | Yes | 253 (76.7) | 66 (73.8) | 1.19 (0.71-2.03) |
|  | No | 77 (23.3) | 24 (26.2) | -------- |
| X-rays during pregnancy in the mother | Yes | 4 (1.0) | 1 (1.1) | 0.94 (0.10-8.52) |
|  | No | 378 (99.0) | 89 (99.9) | -------- |
| Maternal exposure at home before pregnancy | Yes | 133 (35.8) | 25 (27.8) | 1.44 (0.88-2.40) |
|  | No | 239 (64.2) | 65 (72.2) | -------- |
| Maternal exposure at home during pregnancy | Yes | 105 (28.2) | 17 (19.5) | 1.68 (0.95-2.99) |
|  | No | 267 (71.8) | 73 (80.5) | -------- |
| Maternal exposure at home after birth | Yes | 190 (50.1) | 40 (44.5) | 1.25 (0.79-1.99) |
|  | No | 189 (49.9) | 50 (55.5) | -------- |
| Maternal exposure to insecticides after birth | Yes | 145 (38.3) | 29 (32.2) | 1.30 (0.79-2.12) |
|  | No | 234 (61.7) | 61 (67.8) | -------- |
| * IMSS: The Instituto Mexicano del Seguro Social; OR are crude odds ratios | | | | |


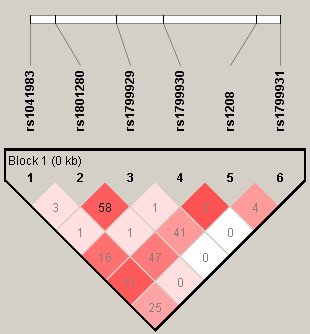


**Supplementary figure 1.** Linkage disequilibrium (LD) map for single nucleotide polymorphisms (SNPs) of *NAT2*. LD plot was generated by Haploview. White bar represents a strand of a chromosome, the black lines on the white bar are SNPs, which are labeled as numbers (1-6) or loci. r2 values is showed into boxes and represents the LD among two SNPs. Low LD between NAT2 SNPS was found.


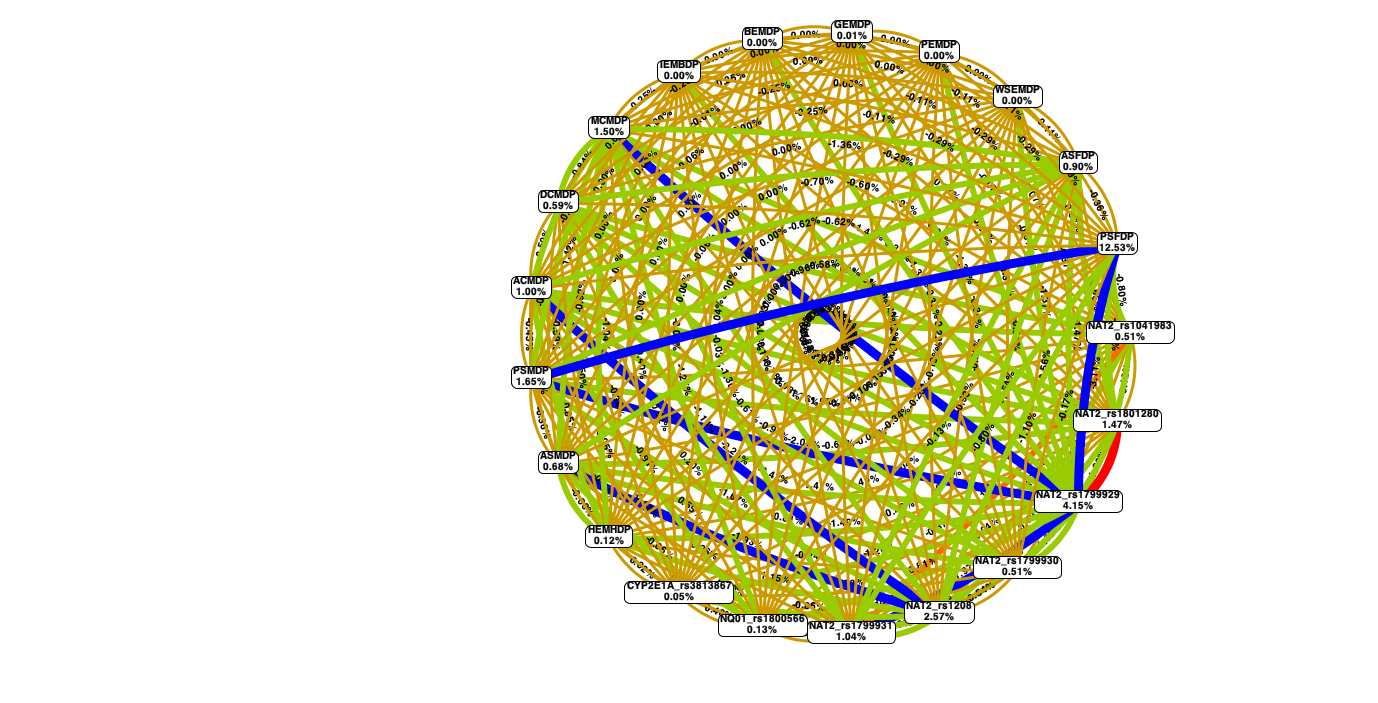

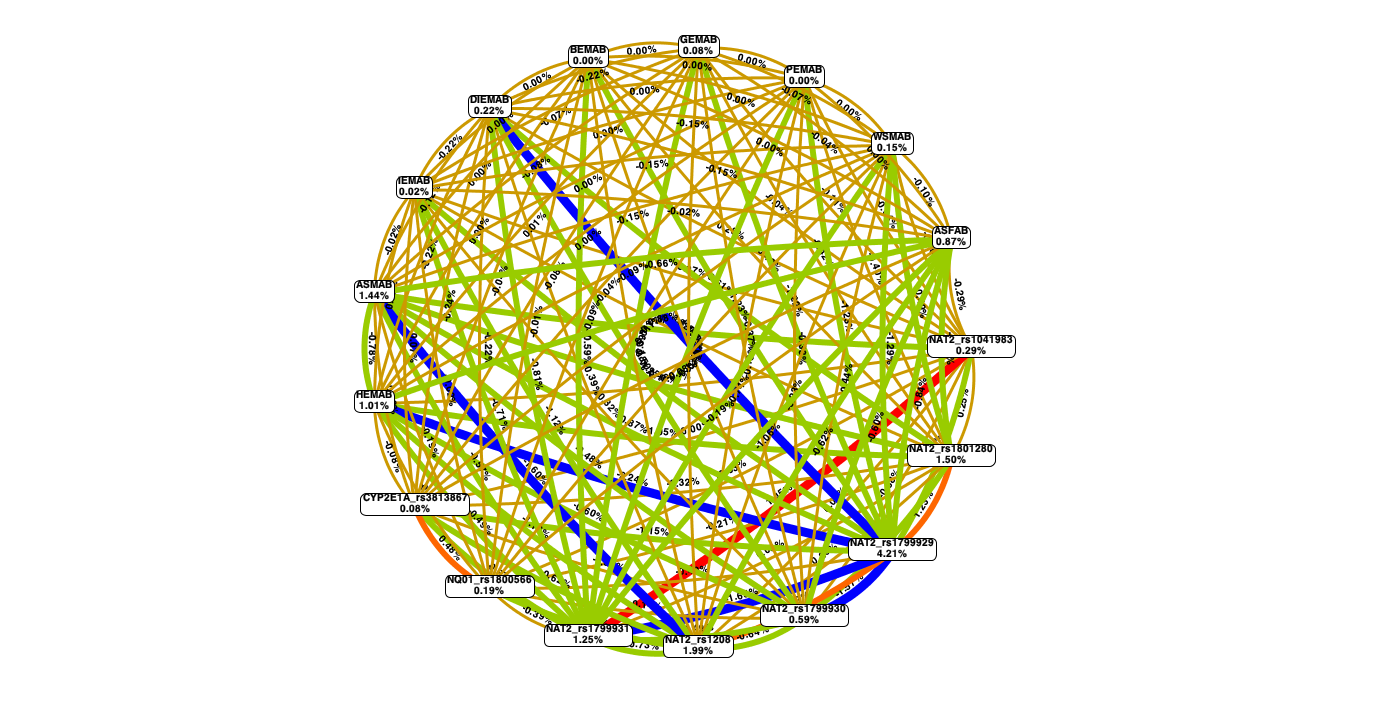


B)

A)

**Supplementary Figure 2.**  Circle graph representing the gene–environmental interactions. **A)** during pregnancy and **B)** after child’s birth. Red lines mean a synergistic effect, blue and green lines represent redundancy and gold lines mean independency. HEMHDP: hydrocarbons exposure by the mother during pregnancy; ASMDP: active smoking by the mother during pregnancy; PSMDP: passive smoking by the mother during pregnancy; ACMDP: alcohol consumption by the mother during pregnancy; DCMDP: drug consumption by the mother during pregnancy; MCMDP: medication consumption by the mother during pregnancy; IEMDP: Insecticide exposure by the mother during pregnancy; BEMDP: benzene exposure by the mother during pregnancy; GEMDP: gasoline exposure by the mother during pregnancy; PEMDP: petroleum exposure by the mother during pregnancy; WSMEDP: wood smoking exposure by the mother during pregnancy; ASFDP: active smoking by the father during pregnancy; PSFDP: passive smoking by the father during pregnancy; HEMAB: hydrocarbons exposure by the mother after child’s birth; ASMAB: active smoking by the mother after child’s birth; IEMAB: insecticide exposure by the mother after child’s birth; DIEMAB: dust insecticide exposure by the mother after child’s birth; BEMAB: benzene exposure by the mother after child’s birth; GEMAB: gasoline exposure after child’s birth; PEMAB: petroleum exposure by the mother after child’s birth; WSMAB wood smoke exposure by the mother after child’s birth; ASFAB: active smoking by the father after child’s birth.


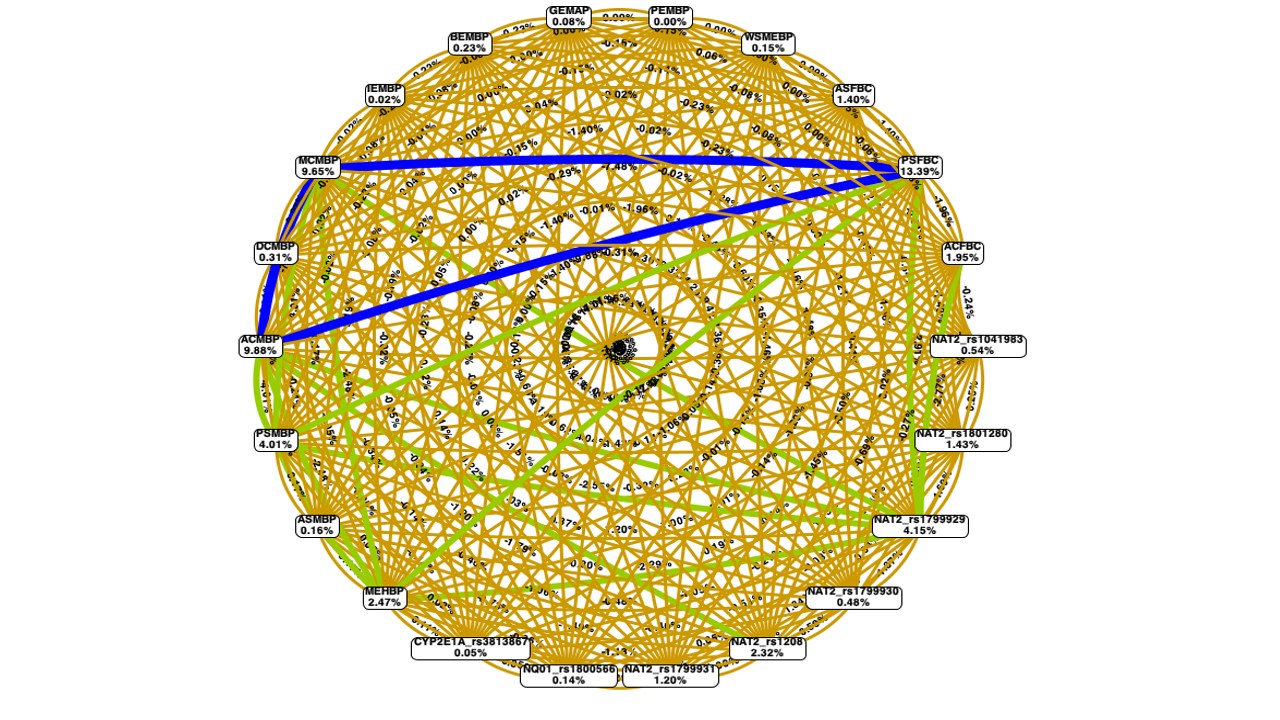


**Supplementary Figure 3**. Circle graph of the MDR analysis of gene-environmental interactions in acute lymphoblastic leukemia. The interaction circle graph was built by gene and xenobiotic factors (boxes) with pairwise connections among them. Values in each box show information gained by each factor, and values and color between nodes are interaction effects. A synergistic or nonadditive effect is represented by positive values and redundancy or correlation is indicated by negative values. Blue lines and green lines indicate redundancy and gold lines mean independency. The passive smoking by the father before conception (PSFBC) and the medication consumption by the mother before pregnancy (MCMBP) have the larger effect on susceptibility to ALL (13.39 and 9.65%, respectively). MEHBP: hydrocarbons maternal exposure at home before pregnancy; ASMBP: active smoking by the mother before pregnancy; PSMBP: passive smoking by the mother before pregnancy; ACMBP: alcohol consumption by the mother before pregnancy; DCMBP: drug consumption by the mother before pregnancy; IEMBP: Insecticide exposure by the mother before pregnancy; BEMBP: benzene exposure by the mother before pregnancy; GEMAP: gasoline exposure by the mother before pregnancy; PEMBP: petroleum exposure by the mother before pregnancy; WSMEBP: wood smoking exposure by the mother before pregnancy; ASFBC: active smoking by the father before conception; ACFBC: alcohol consumption by the father before pregnancy.
